# Stereoselective synthesis of carbocyclic analogues of the nucleoside Q precursor (PreQ_0_)

**DOI:** 10.3762/bjoc.10.135

**Published:** 2014-06-11

**Authors:** Sabin Llona-Minguez, Simon P Mackay

**Affiliations:** 1 Strathclyde Institute of Pharmacy & Biomedical Sciences, University of Strathclyde, 165 Cathedral Street, Glasgow, G4 0RE, United Kingdom; 2 Science for Life Laboratory, Division of Translational Medicine & Chemical Biology, Department of Medical Biochemistry & Biophysics, Karolinska Institutet, Stockholm, S-171 21, Sweden

**Keywords:** diol synthesis, nucleoside, PreQ_0_, stereoselective amine synthesis, triol synthesis

## Abstract

A convergent and stereoselective synthesis of chiral cyclopentyl- and cyclohexylamine derivatives of nucleoside Q precursor (PreQ_0_) has been accomplished. This synthetic route allows for an efficient preparation of 4-substituted analogues with interesting three-dimensional character, including chiral cyclopentane-1,2-diol and -1,2,3-triol derivatives. This unusual substitution pattern provides a useful starting point for the discovery of novel bioactive molecules.

## Introduction

**Figure 1:**
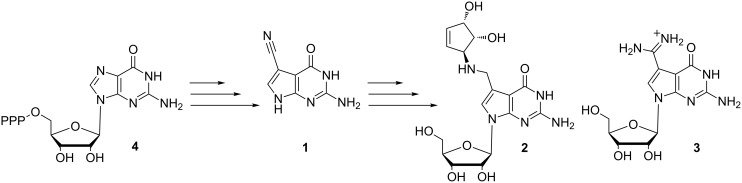
Biosynthetic pathway leading to nucleosides queuosine and archaeosine.

7-Deazapurine (pyrrolo[2,3-*d*]pyrimidine) nucleosides are commonly found in nature playing a variety of roles such as building blocks of nucleic acids and tRNA, metabolites or antimetabolites [1]. Deazapurine ribonucleosides also show interesting pharmacological profiles including antibacterial, antiviral and anticancer properties [2–4]. Nucleoside Q precursor (PreQ_0_) **1** is a common precursor in the biosynthesis of queuosine (Q, **2**) and archaeosine (G^+^, **3**), two hyper-modified nucleosides present in the tRNA of prokaryote/eukaryote and euryarchaeota, respectively [5–6]. In turn, the biosynthesis of PreQ_0_ originates from guanosine 5’-triphosphate (GTP, **4**) [7] (Figure 1) and involves four steps via a tetrahydropterine intermediate.

The pyrrolo[2,3-*d*]pyrimidine core is a privileged scaffold for the development of kinase inhibitors; an inspection of the medicinal chemistry literature reveals >200 publications in the field. Additionally, PreQ_0_ meets all the criteria dictated by the “2-0” rule of kinase-likeness proposed by Aronov et al. [8]. It is likely that compounds derived from PreQ_0_ display kinase activity.

**Figure 2:**
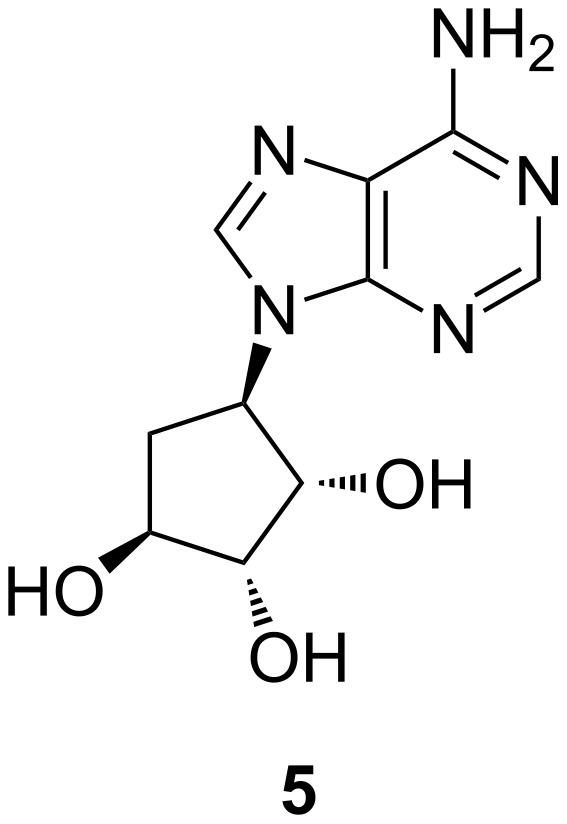
Chemical structure of noraristeromycin.

7-Deazapurine nucleoside chemistry has been the subject of extensive study [1] and several syntheses of the PreQ_0_ base or ribonucleoside [9–16] and queuosine [17] have been reported in the literature. Despite this long-lasting interest, examples of purine-based nucleosides containing a sugar or carbosugar motif at the 4-position of the heterocyclic core (systematic numbering) are scarce in the chemical literature and the methods available generally lack experimental information, making them unsatisfactory [18–25]. Inspired by the cyclopentane-1,2,3-triol motif present in noraristeromycin **5** (Figure 2), an IκB kinase inhibitor with antiviral and anti-inflammatory activity [26–27], we decided to investigate a synthetic route that would allow for the incorporation of carbocyclic systems with interesting three-dimensional character at the 4-position of PreQ_0_ as part of our fragment-based kinase inhibitor library generation programme.

## Results and Discussion

**Figure 3:**
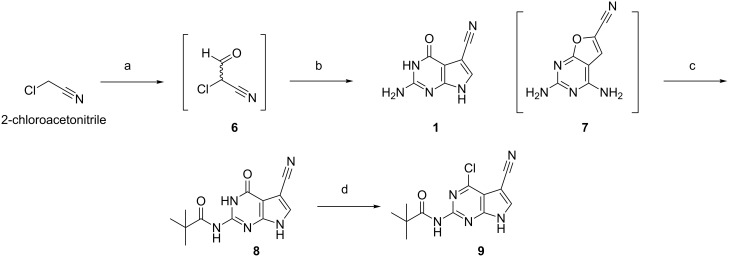
Synthesis of PreQ_0_ and chloro-intermediate **9**. Reagents and conditions: (a) Methyl formate, NaOMe, PhMe, 3 h, 0 °C; (b) 2,6-diaminopyrimidin-4(3*H*)-one, NaOAc, H_2_O, 17 h, 100 °C, 60% (over two steps); (c) PivCl, pyridine, 2 h, 85 °C, 64%; (d) POCl_3_, DMA, BnEt_3_NCl, MeCN, 1 h, 90 °C, 35%.

Our retrosynthetic approach introduces the diversity point at a late stage and takes advantage of the heterocyclic lactam present in PreQ_0_ after activation and subsequent nucleophilic aromatic substitution. This convergent synthesis allowed us to prepare diverse chiral amine building blocks and react them with a common halo-purine intermediate to obtain the desired final products. The pyrrolo[2,3-*d*]pyrimidine core of PreQ_0_ was furnished following a method described by Klepper et al. [13] (Figure 3). The two step process started with the formylation of chloroacetonitrile with methyl formate. The resulting volatile and unstable chloroaldehyde **6** was used without further purification. Cyclocondensation of **6** with 2,4-diamino-4-hydroxypyrimidine afforded **1** regiospecifically with no detectable formation of the undesired 6-substituted-furo[2,3-*d*]pyrimidine **7**. Direct chlorination of **1** in a moderate scale (1 g) using POCl_3_ proved to be very low yielding [28]. It remains unclear if this was due to the poor solubility of PreQ_0_ or to the presence of unprotected amino functionalities. In order to overcome this issue, the exocyclic amine was protected [14] (Figure 3). The resulting pivalamide **8** proved to be more soluble than **1** and the subsequent halogenation step was accomplished in the presence of a phase transfer catalyst, affording the desired chloro-intermediate **9** in fair yield. In our hands, nucleophilic aromatic substitution on **9** using amines of diverse nature usually proceeds smoothly and allows for a clean pivalamide deprotection [29]. For this reason we decided to couple the chiral amines of interest and remove protecting groups in a one-pot procedure.

**Figure 4:**
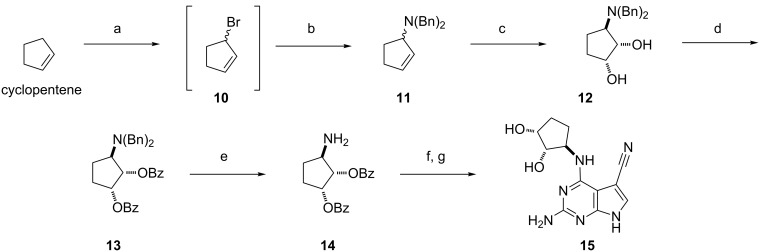
Synthesis of **15**, a (1*RS*,2*SR*,3*RS*)-3-aminocyclopentane-1,2-diol derivative of PreQ_0_. Reagents and conditions: (a) NBS, (PhCO_2_)_2_, CCl_4_, 1 h, 90 °C; (b) NH(Bn)_2_, CCl_4_, 12 h, rt, 70% (over two steps); (c) OsO_4_, NMO, acetone/H_2_O, 4 h, rt, 72%, 96% ds; (d) BzCl, pyr, 24 h, 0 °C to rt, 84%; (e) H_2_ (1 atm), Pd(OH)_2_, EtOH/EtOAc, 16 h, rt, 98%; (f) **9**, Et_3_N, *n*-BuOH, 16 h, 130 °C; (g) KOH, *n*-BuOH/EtOH, 16 h, 80 °C, 42%.

First we investigated a more synthetically accessible (1*RS*,2*SR*,3*RS*)-3-aminocyclopentane-1,2-diol core. Our previous experience in coupling diols and triols at high temperatures with chloro-intermediate **9** showed that more than one unprotected alcohol functionality leads to complex reaction mixtures and very low yields of isolated products [29], hence we protected all hydroxy groups as esters. We chose the benzoate protecting group to generate UV–visible intermediates and because its ease of cleavage under basic conditions would converge with the final pivalamide deprotection step. We adapted this protecting group strategy to Bond’s synthetic route since it was the most concise and diastereospecific available [30] (Figure 4). The process started with a Wohl–Ziegler allylic bromination of cyclopentene. The volatile and unstable allylic halide **10** was immediately reacted with excess *N*,*N*-dibenzylamine and the resulting allylic amine **11** was obtained in good yield over two steps. Next, we introduced the two hydroxy groups *trans*- to the amine moiety using an Upjohn dihydroxylation. Freshly-prepared aqueous OsO_4_ stock solutions were required to obtain good yields in this step. The reaction proceeded smoothly and the ^1^H NMR spectra of the crude reaction mixture showed a 96:4 ratio of *cis*- to *trans*-isomers. After column chromatography the isolated diol **12** showed a diastereomeric purity of >99% by ^1^H NMR. The dibenzoate **13** was obtained in good yield following standard acylation conditions [31]. Final removal of the two benzyl groups was accomplished in excellent yield using catalytic hydrogenation [30], using EtOAc as a co-solvent to improve the substrate solubility. Amine **14** was coupled with **9** and the pivalamide and benzoate groups were cleaved in the one-pot procedure previously described to afford **15**, the (1*RS*,2*SR*,3*RS*)-3-aminocyclopentane-1,2-diol derivative of PreQ_0_.

**Figure 5:**
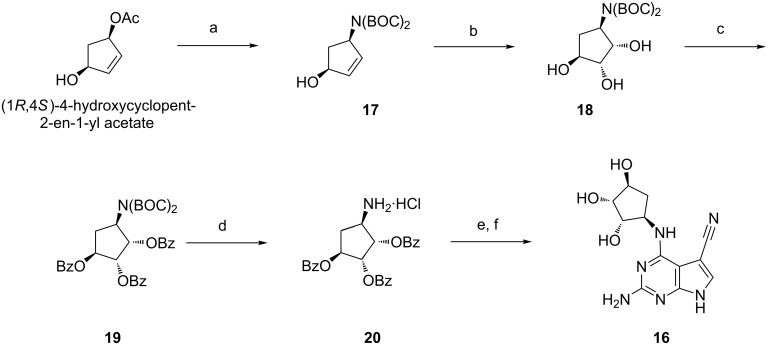
Synthesis of **16**, a (1*S*,2*R*,3*S*,4*R*)-4-aminocyclopentane-1,2,3-triol derivative of PreQ_0_. Reagents and conditions: (a) Pd(PPh_3_)_4_, PPh_3_, NaH, NH(Boc)_2_, THF/DMF, 1 day, 50 °C, 42%; (b) OsO_4_, NMO, acetone/H_2_O, 1 day, rt, 83%; (c) BzCl, pyr, 17 h, 0 °C to rt, 74%; (d) 4 M HCl in 1,4-dioxane, 16 h, 0 °C to rt, 76%; (e) **9**, Et_3_N, *n*-BuOH, 16 h, 130 °C; (f) KOH, *n*-BuOH/EtOH, 16 h, 80 °C, 33%.

Adapting a protocol developed by Springthorpe et al. [32], we then investigated a route to prepare the enantiopure (1*S*,2*R*,3*S*,4*R*)-4-aminocyclopentane-1,2,3-triol analogue of PreQ_0 _**16** (Figure 5)_._ The first step is a Tsuji–Trost allylation of sodium di-*tert*-butyliminodicarboxylate. The reaction proceeded with an overall retention of configuration as expected and the ^1^H NMR spectra of the crude reaction mixture only showed the desired diastereomer **17**. Several known catalytic systems were tested [29]: Pd(PPh_3_)_4_/PPh_3_ in THF/DMF [33], Pd_2_(dba)_3_/diphos in THF/DMF [34], Pd_2_(dba)_3_/dppf in THF [35]. The first set of conditions proved to be the most successful, although addition of DMF was required to improve the solubility of the reactants. It is worth noting that this reaction proved to be extremely sensitive to the presence of moisture and oxygen. The bulky nature of the nucleophile used aided in the diastereoselectivity of the following *syn*-dihydroxylation. Using the Upjohn conditions previously described we obtained the desired triol **18** in good yield and excellent diastereoselectivity (>99% by ^1^H NMR after column chromatography) [30]. Tri-benzoate **19** was subsequently obtained in good yield using the standard benzoylation conditions [31]. Final removal of the two BOC protecting groups using 4 M HCl in 1,4-dioxane yielded amine **20** as the hydrochloride salt. Amine **20** was coupled with chloro-intermediate **9** and the remaining four protecting groups were cleaved in a one-pot procedure under basic conditions, generating the desired triol **16**.

**Figure 6:**
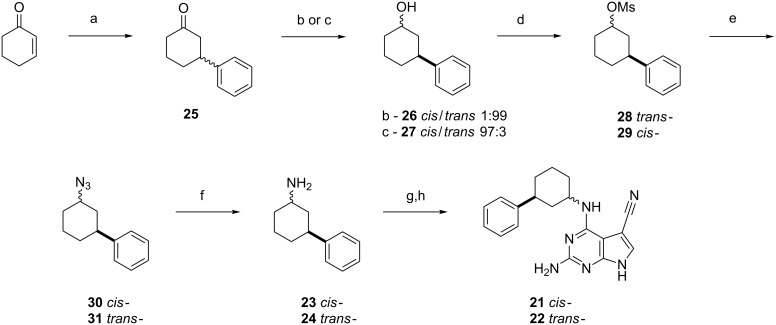
Synthesis of **21** and **22**, 3-arylcyclohexylamine derivatives of PreQ_0._ Reagents and conditions: (a) PhB(OH)_2_, Pd(OAc)_2_, bpy, H_2_O/THF/AcOH, 3 days, 60 °C, 98%; (b) K-Selectride, THF, 2 h, −90 °C; then KOH (aq), H_2_O_2_, 30 min, rt, 80%, 99% ds; (c) LiAlH_4_, Et_2_O, 1,5 h, 0 °C to rt; then NaOH (aq), 30 min, rt, 76%, 97% ds (d) MsCl, Et_3_N, THF, 16 h, rt, (*trans*- 67%, *cis*- 64%); (d) NaN_3_, DMF, 2 days, 80 °C, (*trans*- 56%, *cis*- 67%); (f) HCO_2_NH_4_, Pd/C, MeOH, 1.5 h, reflux, (*trans*- 92%, *cis*- 94%); (g) **9**, Et_3_N, *n*-BuOH, 16 h, 130 °C; (h) KOH, *n*-BuOH/EtOH, 16 h, 80 °C, (*trans*- 38%, *cis*- 50%).

To extend into hydrophobic chemical space around our PreQ_0_ analogues, we prepared two novel derivatives containing the unusual 3-arylcyclohexylamine chiral motif present in **21** and **22**. Zhou et al. had reported an asymmetric synthesis leading to the *cis*-3-arylcyclohexanamines with reasonable diastereoselectivity [36], but since initially we did not require an enantioselective synthesis and the Zhou method employed rather expensive reagents, we investigated a simpler and cheaper route to access both *cis*- and *trans*-isomers. We envisioned a stereoselective synthesis that would potentially allow for the introduction of diverse aryl groups at the 3-position of the cyclohexane ring using commercially available arylboronic acids as building blocks, and Pd catalysis to form the new C–C bond, followed by a highly diastereoselective ketone-to-amine conversion. Others have reported on similar preparations of 3-phenylcyclohexanamines, although with poor diastereomeric control [37–38]. 1-Cyclohex-2-enone provided the two required synthetic handles: a sp^2^ carbon for Pd chemistry and a ketone for further derivatization into an amine group (Figure 6). The synthesis of *cis*- and *trans*-3-arylcyclohexylamines **23** and **24** started with a Pd^(II)^-catalyzed Miyaura 1,4-conjugate addition of phenylboronic acid to cyclohexenone [39]. The resulting ketone **25** was reduced to the axial [40–41] and equatorial [40] alcohols **26** and **27** with excellent diastereoselectivity thanks to steric control of the hydride source. After column chromatography both alcohols showed a diastereomeric purity of >99% by ^1^H NMR. Mitsunobu reaction on the secondary alcohols using DEAD or DIAD did not provide the desired azides [42–43] nor did a one-pot Appel reaction/nucleophilic substitution/Staudinger reaction protocol involving a double inversion of configuration [44]. Mesylation of **26** and **27** lead to intermediates **28** and **29** [45], which were subsequently reacted with sodium azide inverting the stereochemistry as required [46]. A final transfer hydrogenation of **30** and **31** yielded the desired amines rapidly and with excellent yields [47]. Amines **23** and **24** were reacted with chloro-intermediate **9** and the pivalamide groups were cleaved under basic hydrolysis conditions to yield **21** and **22**.

## Conclusion

In conclusion, a concise and stereoselective synthesis of novel cyclopentyl and cyclohexyl analogues of PreQ_0_ has been developed to expand our fragment-based kinase library. This synthetic protocol involves asymmetric syntheses of hydroxy-protected (1*RS*,2*SR*,3*RS*)-3-aminocyclopentane-1,2-diol and (1*S*,2*R*,3*S*,4*R*)-4-aminocyclopentane-1,2,3-triol or *cis*- and *trans*-3-arylcyclohexylamines, which are in turn reacted with a conveniently PreQ_0_-derived halo-intermediate and subsequently deprotected in a one-pot fashion. Pharmacological assessment of these novel PreQ_0_ derivatives is currently underway in a variety of kinase-inhibitory studies and will be reported in due course.

## Supporting Information

File 1 General methods, experimental procedures and copies of ^1^H/^13^C NMR spectra and HPLC UV traces of final compounds **15**, **16**, **21** and **22**.
