# Supplementary material for: Stereoselective synthesis of carbocyclic analogues of the nucleoside Q precursor (PreQ0)
Source: Beilstein J Org Chem. 2014 Jun 11;10:1333–8. doi: 10.3762/bjoc.10.135 (PMC4077357; doi:10.3762/bjoc.10.135)
Supplement: File 1 — General methods, experimental procedures and copies of 1H/13C NMR spectra and HPLC UV traces of final compounds 15, 16, 21 and 22. [file Beilstein_J_Org_Chem-10-1333-s001.pdf]

Supporting Information

for

Stereoselective synthesis of carbocyclic analogues of the nucleoside Q precursor ($PreQ_0$)

Sabin Llona-Minguez*[,1,2] and Simon P Mackay*[,1]

Address: [1]Strathclyde Institute of Pharmacy & Biomedical Sciences, University of Strathclyde, 165 Cathedral Street, Glasgow, G4 0RE, United Kingdom and [2]current address: Science for Life Laboratory, Division of Translational Medicine & Chemical Biology, Department of Medical Biochemistry & Biophysics, Karolinska Institutet, Stockholm, S-171 21, Sweden

Email:
Sabin Llona-Minguez - sabin.llona.minguez@scilifelab.se; Simon P Mackay - simon.mackay@strath.ac.uk

*Corresponding author

General methods, experimental procedures and copies of ^{1}H / ^{13}C NMR spectra and HPLC UV traces of final compounds 15, 16, 21 and 22.

General methods

Solvents (reagent grade or better) were purchased from Sigma-Aldrich or Fischer Scientific. Anhydrous solvents where purchased from Sigma-Aldrich. Deuterated solvents were purchased from Sigma-Aldrich. Chemicals (95% purity or above) were purchased from Acros Organics, Alfa Aesar, Apollo, Fluorochem or Sigma-Aldrich. Solvents and chemicals were used as received without further purification or treatment. Oxygen- or moisture-sensitive reactions were carried out under positive nitrogen atmosphere.

The progress of the reactions was monitored on Merck 60F254 TLC plates. Spots were visualized by irradiation with ultraviolet light (254/366 nm) or $KMnO_4$, ninhydrin or phosphomolybdic acid (PMA) TLC stains. Column chromatography was performed with a Biotage SP4 MPLC system (unless stated otherwise); cartridge size and eluent specified in the corresponding experiments (% is referring to the most polar solvent in the mixture); using silica gel as the stationary phase (particle size 0.040–0.063 mm, Merck or Fisher Scientific).

Specific rotations were measured in a Perkin Elmer Polarimeter 341 apparatus at 20 °C and a wavelength of 589 nm (sodium D line) in DMSO UV spectrophotometric analysis grade.

Melting points were taken in open capillaries on a Stuart Scientific Melting point SMP1 apparatus in Celsius degrees (°C) and were uncorrected. Decomposition temperature indicated as (d).

FTIR analyses were performed on a Thermo Scientific Nicolet iS10. Samples were measured neat and frequencies are expressed as cm^{-1}.

^{1}H and ^{13}C NMR data were recorded on either a JEOL ECX-400 (400 MHz) or Bruker Avance3/DPX400 (400 MHz) spectrometers at 400.0 and 100.6 MHz respectively. Chemical shifts (δ) are expressed in parts per million (ppm) coupling constants (*J*) are in hertz (Hz). Chemical shifts (δ) are reported relative to TMS (δ = 0 ppm) and/or referenced to the solvent in which they were measured. All measurements were carried out at 298 K (except when stated). Abbreviations used in the description of NMR data are as follows: app, apparent; s, singlet; bs, broad singlet; d, doublet; t, triplet; q, quartet; p, pentuplet; m, multiplet.

HRMS was conducted using a Thermo Scientific Exactive Orbitrap Mass Analyzer. LR-MS was conducted using a ThermoQuest Finnigan LCQ Duo instrument. GC–MS was conducted using a ThermoQuest Finnigan Polaris Q instrument.

Final compounds possessed a purity of ≥95% by HPLC analysis conducted using an Agilent Technologies 1220 series system (Methods A and B). Column: Agilent Eclipse XDB C18 4.6 mm ID × 250 mm (5µm) 80 Å. Flow rate: 1 mL/min. Detector: 254 nm. Sample volume: 10 µL. Mobile phase: **Method A**) 15% MeCN in H_2O (3 min), 15 to 90% MeCN in H_2O (12 min) followed by equilibration / blank run; **Method B**) 5% MeCN in H_2O (3 min), 5 to 100% MeCN in H_2O (14 min), 100% MeCN in H_2O (5 min), 100 to 5% MeCN in H_2O (5 min), 5% MeCN in H_2O (5 min) followed by blank run; Flow rate: 0.4 mL/min. Detector: 254 nm.

Experimental procedures

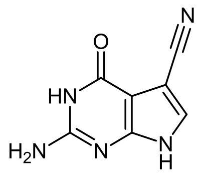

2-Amino-4-oxo-4,7-dihydro-3*H*-pyrrolo[2,3-*d*]pyrimidine-5-carbonitrile (1) [1]

Methyl formate (18.0 mL, 17.48 g, 291.4 mmol) in toluene (8 mL) was added at 0 °C to a stirred suspension of NaOMe (14.30 g, 264.9 mmol) in toluene (200 mL). This was followed by dropwise addition of chloroacetonitrile (16.8 mL, 20.00 g, 264.9 mmol) in toluene (60 mL) over 1 h. The reaction mixture was stirred for 3 h followed by addition of H_2O (150 mL). The organic layer was separated and the aqueous layer was acidified to pH 5 using 6 M HCl and subsequently extracted with EtOAc (3 × 100 mL). The organic layers were combined and dried over $MgSO_4$ and concentrated in vacuo (40 °C, 70 mbar). The dark residue was suspended in H_2O (60 mL) and added to a solution of NaOAc (16.39 g, 199.8 mmol) and 2,6-diaminopyrimidin-4(3*H*)-one (12.00 g, 95.2 mmol) in H_2O (200 mL) (previously stirred at 100 °C until complete dissolution). The reaction was refluxed for 16 h. After cooling to room temperature the suspension was filtered and washed with H_2O (2 × 20 mL), acetone (2 × 10 mL) and Et_2O (2 × 40 mL) to yield **1** (10.11 g, 60%) as a light tan solid. ^{1}H NMR (400 MHz, DMSO-d_6) δ: 6.37 (bs, 2H), 7.58 (s, 1H), 10.70 (bs, 1H), 11.95 (bs, 1H); ^{13}C NMR (100 MHz, DMSO-d_6) δ: 86.0, 99.2, 116.3, 128.2, 158.0, 152.1, 154.2.

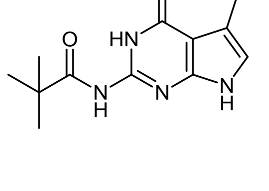

***N*-(5-Cyano-4-oxo-4,7-dihydro-3*H*-pyrrolo[2,3-*d*]pyrimidin-2-yl)pivalamide (8)** [2]

1 (7.33 g, 41.85 mmol) was stirred in dry pyridine (60 mL) at 60 °C for 1 h. Upon formation of a homogenous suspension, the mixture was cooled to 0 °C and treated dropwise with pivaloyl chloride (10.5 mL, 10.23 g, 84.87 mmol). The suspension was then stirred at 85 °C for 2 h. After cooling to room temperature, the resulting suspension was neutralized with 33% ammonia in H_2O and left to stand at 4 °C for 16 h. The suspension was filtered off, washed with H_2O (10 mL), dried and then triturated with Et_2O (2 × 10 mL) to afford **8** (6.97 g, 64%)

as a pale brown solid. ^{1}H NMR (400 MHz, DMSO-d_6) δ: 1.25 (s, 9H), 7.93 (s, 1H), 11.00 (bs, 1H), 12.11 (bs, 1H) and 12.65 (bs, 1H); ^{13}C NMR (100 MHz, DMSO-d_6) δ: 27.0, 40.7, 86.9, 103.7, 115.9, 130.9, 149.1, 149.1, 156.4, 181.7.

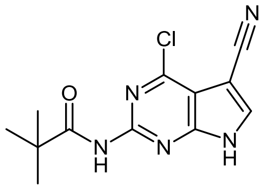

***N*-(4-Chloro-5-cyano-7*H*-pyrrolo[2,3-*d*]pyrimidin-2-yl)pivalamide (9)** [2]

A suspension of **8** (6.82 g, 26.31 mmol), *N*,*N*-dimethylaniline (14 mL, 13.39 g, 110.48 mmol) and triethylbenzylammonium chloride (2.93 g, 13.15 mmol) in dry MeCN (100 mL) was treated dropwise with $POCl_3$ (24.5 mL, 40.33 g, 263.05 mmol). The reaction mixture was refluxed for 1 h, allowed to cool down and concentrated in vacuo. The resulting dark oil was cautiously treated with ice and was set to pH = 5 using 33% ammonia in H_2O. The aqueous layer was extracted with EtOAc (4 × 100 mL) and the combined organic layers were dried over $MgSO_4$ and concentrated in vacuo. The residue was triturated with Et_2O (3 × 30 mL), MeOH (2 × 20 mL) and Et_2O (2 × 20 mL) to give **9** (2.56 g, 35%) as a light tan solid. ^{1}H NMR (400 MHz, DMSO-d_6) δ: 1.22 (s, 9H), 8.50 (s, 1H), 10.27 (bs, 1H), 13.35 (bs, 1H); ^{13}C NMR (100 MHz, DMSO-d_6) δ: 27.4, 40.3, 84.1, 111.8, 115.1, 138.2, 151.4, 153.5, 153.8, 176.5.

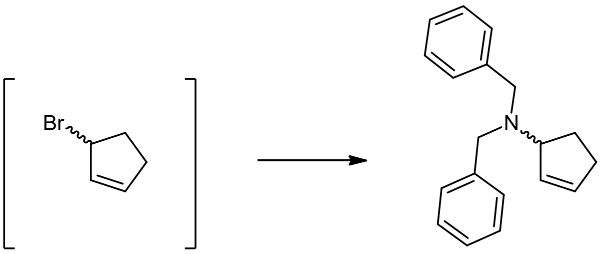

3-(*N*,*N*-Dibenzylamino)cyclopent-1-ene (11) [3]

A mixture of cyclopentene (12.3 mL, 9.18 g, 0.135 mol), NBS (6.01 g, 33.71 mmol) and benzoyl peroxide (70%, 163 mg, 0.67 mmol) in CCl_4 (21 mL) was heated at reflux for 1 h. The reaction mixture was cooled to 0 °C, filtered through a pad of Celite® (eluent CCl_4) and solvent and cyclopentene were distilled off in vacuo. The residue was dissolved in CCl_4 (30 mL), cooled to 0 °C and *N*,*N*-dibenzylamine (16.2 mL, 16.60 g, 84.28 mmol)

was added to the crude solution of bromide **10**. The mixture then warmed to room temperature and stirred for 30 min. The reaction mixture was then filtered, heated to 40 °C, and stirred at this temperature for 1 h, then filtered and stirred at room temperature for 16 h. The mixture was then filtered and concentrated in vacuo. Chromatographic purification (Biotage SP4, 100 g cartridge, solvent system: 10% Et_2O in Hex/Hex, gradient: 0% 4CV; 0-10% 10CV) yielded **11** (6.230 g, 70%) as a colourless oil. ^{1}H NMR (400 MHz, $CDCl_3$) δ: 1.78-1.93 (m, 2H), 2.22-2.29 (m, 1H), 2.34-2.41 (m, 1H), 3.43 (d, *J* = 13.8 Hz, 2H), 3.64 (d, *J* = 13.8 Hz, 2H), 4.03-4.05 (m, 1H), 5.75-5.76 (m, 1H), 5.86-5.89 (m, 1H), 7.19-7.23 (m, 2H), 7.27-7.31 (m, 4H), 7.37-7.39 (m, 4H); ^{13}C NMR (100 MHz, $CDCl_3$) δ: 23.4, 31.9, 54.5, 66.1, 126.8, 128.3, 128.8, 132.1, 133.3, 140.8.

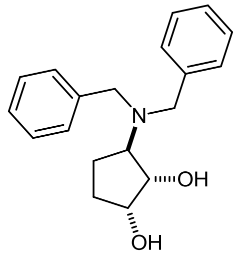

(1*RS*,2*SR*,3*RS*)-3-(Dibenzylamino)cyclopentane-1,2-diol (12) [3]

A solution of OsO_4 in H_2O (4% w/v, 0.32 mL, 13 mg, 0.05 mmol) was added to a stirred solution of **11** (1.31 g, 4.98 mmol) and NMO (1.33 g, 14.94 mmol) in acetone/H_2O (4:1, 35 mL) and the resultant mixture was stirred at room temperature for 4 h. Sat. aq. Na_2SO_3 (5 mL) was then added and the solution was stirred for an additional 30 min. Acetone was evaporated in vacuo, H_2O (10 mL) was added and the aqueous layer was extracted with DCM (3 × 20 mL). The organic layer was adsorbed on silica gel and concentrated in vacuo. Chromatographic purification (Biotage SP4, 50g cartridge, solvent system: EtOAc/Hex, gradient: 10% 4CV; 10–20% 6CV; 20% 4CV; 20–60% 6CV; 60% 6CV) yielded **12** (1.06 g, 72%) as a white solid. ^{1}H NMR (400 MHz, $CDCl_3$) δ: 1.52-1.75 (m, 2H), 1.83-1.96 (m, 2H), 2.28 (bs, 1H), 2.37 (bs, 1H), 3.27 (app dt, *J* = 8.6, 8.4 Hz, 1H), 3.57 (d, *J* = 13.9 Hz, 2H), 3.78 (d, *J* = 13.9 Hz, 2H), 3.92 (app dd, *J* = 8.2, 5.2 Hz, 1H), 3.99-4.05 (m, 1H), 7.21-7.25 (m, 2H), 7.29-7.32 (m, 4H), 7.35-7.37 (m, 4H); ^{13}C NMR (100 MHz, $CDCl_3$) δ: 19.6, 29.0, 55.0, 65.1, 71.1, 74.6, 127.1, 128.4, 128.7, 139.9; HRMS (ESI) calcd for $C_{19}H_{24}O_2N$ $[M+H]^+$: 298.1802, found: 298.1798.

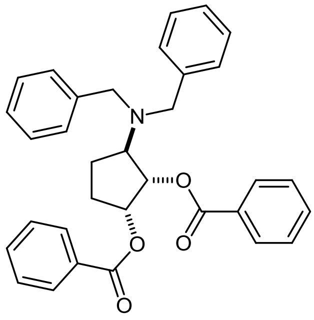

(1*RS*,2*SR*,3*RS*)-3-(Dibenzylamino)cyclopentane-1,2-diyl-dibenzoate (13)

Benzoyl chloride (1.62 ml, 1.96 g, 13.92 mmol) was added dropwise to a stirred solution of **12** (1.03 g, 3.48 mmol) in dry pyridine (30 mL) at 0 °C, left to warm to room temperature and stirred for 1 day. Pyridine was evaporated in vacuo and the residue was suspended in Et_2O (40 mL), cooled to 0 °C followed by addition of saturated aqueous $NaHCO_3$ (10 mL). The organic layer was then washed with saturated aqueous $NaHCO_3$ (3 × 20 mL) and subsequently adsorbed on silica gel and concentrated in vacuo. Chromatographic purification (Biotage SP4, 50g cartridge, solvent system: 10% Et_2O in Hex/Hex, gradient: 0% 3CV; 0–10% 3CV; 10% 4CV; 10–20% 4CV; 20% 4CV; 20–40% 6CV) yielded **13** (1.45 g, 84%) as a white solid. ^{1}H NMR (400 MHz, $CDCl_3$) δ: 1.75-1.84 (m, 1H), 1.88-1.96 (m, 1H), 2.01-2.10 (m, 1H), 2.18-2.26 (m, 1H), 3.67 (d, *J* = 13.8 Hz, 2H), 3.77 (d, *J* = 13.8 Hz, 2H), 3.82 (app dt, *J* = 8.6, 8.4 Hz, 1H), 5.54 (dt, *J* = 5.0, 4.6 Hz, 1H), 5.67 (dd, *J* = 7.9, 5.2 Hz, 1H), 7.20-7.40 (m, 14 H), 7.49 (m, 1H), 7.55 (m, 1H), 7.76 (dd, *J* = 8.4, 1.3 Hz, 2H), 7.94 (dd, *J* = 8.4, 1.3 Hz, 2H); ^{13}C NMR (100 MHz, $CDCl_3$) δ: 20.7, 27.6, 54.7, 62.2, 73.3, 74.0, 126.9, 128.3, 128.3, 128.4, 128.7, 129.6, 129.8, 130.1, 130.2, 132.9, 133.0, 139.7, 165.7, 165.9; HRMS (ESI) calcd for $C_{33}H_{32}O_4N$ $[M+H]^+$: 506.2326, found: 506.2321.

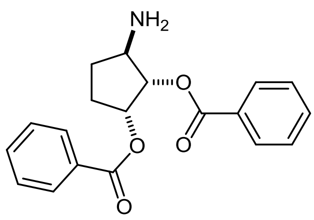

(1*RS*,2*SR*,3*RS*)-3-Aminocyclopentane-1,2-diyl dibenzoate (14)

$Pd(OH)_2/C$ (20% w/w, 700 mg) was added to a vigorously stirred solution of **13** (1.42 g, 2.81 mmol) in EtOH/EtOAc (5:1, 60 mL) and the resultant suspension was stirred at room temperature under H_2 (1 atm) for

16 h. The suspension was filtered through a Celite® pad (eluent MeOH) and concentrated in vacuo. The residue was triturated with Et_2O (2 × 20 mL) to give **14** (908 mg, 98%) as a white solid. ^1H NMR (400 MHz, DMSO-d_6) δ: 1.73-1.82 (m, 1H), 1.93-2.02 (m, 1H), 2.32-2.43 (m, 2H), 3.93 (app dt, *J* = 8.0, 7.7 Hz, 1H), 5.49 (dd, *J* = 7.4, 5.1 Hz, 1H), 5.59-5.63 (m, 1H), 7.42-7.49 (m, 4H), 7.61-7.66 (m, 2H), 7.86-7.89 (m, 2H), 8.38 (bs, 2H); ^{13}C NMR (100 MHz, DMSO-d_6) δ: 25.2, 27.7, 53.4, 73.2, 76.6, 129.1, 129.2, 129.6, 129.7, 129.8, 129.9, 134.1, 134.1, 165.4, 165.5; HRMS (ESI) calcd for $C_{19}H_{20}O_4N$ $[M+H]^+$: 326.1387, found: 326.1382.

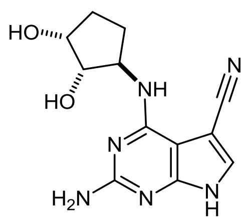

2-Amino-4-((1*RS*,2*SR*,3*RS*)-2,3-dihydroxycyclopentylamino)-7*H*-pyrrolo[2,3-*d*]pyrimidine-5-carbonitrile (15)

9 (80 mg, 0.29 mmol), **14** (70 mg, 0.35 mmol) and Et_3N (0.04 mL, 29 mg, 0.29 mmol) were suspended in *n*-BuOH (4 mL) and refluxed for 16 h. After cooling to room temperature EtOH (4 mL) and KOH (3 pellets) were added and the reaction mixture was heated at 80 °C for 20 h. Then pH was neutralized using 6 N HCl and subsequently concentrated in vacuo. The remaining solid was suspended in MeOH, adsorbed on silica gel and concentrated in vacuo. Chromatographic purification (Biotage SP4, 50 g cartridge, solvent system: MeOH/$CHCl_3$, gradient: 0% 4CV; 0-15% 14CV; 15% 4CV) yielded **15** (33 mg, 42%) as a white solid. Mp: 252-254 °C (d); ^1H NMR (400 MHz, DMSO-d_6) δ: 1.29-1.38 (m, 1H), 1.47-1.55 (m, 1H), 1.87-1.96 (m, 1H), 2.18-2.27 (m, 1H), 3.74 (app dt, *J* = 8.0, 5.0 Hz, 1H), 3.91 (app dt, *J* = 8.8, 4.4 Hz, 1H), 4.32 (app dt, *J* = 15.7, 7.9 Hz, 1H), 4.39 (d, *J* = 3.3 Hz, 1H), 4.94 (d, *J* = 5.5 Hz, 1H), 5.66 (d, *J* = 7.1 Hz, 1H), 5.99 (bs, 2H), 7.69 (s, 1H), 11.83 (bs, 1H); ^{13}C NMR (100 MHz, DMSO-d_6) δ: 27.0, 28.8, 55.4, 70.6, 77.8, 81.5, 94.2, 116.9, 128.4, 153.0, 156.5, 160.8; HRMS (ESI) calcd for $C_{12}H_{13}O_2N_6$ $[M+H]^+$: 273.1105, found: 273.1107; HPLC t_R = 4.47 min (Method A).

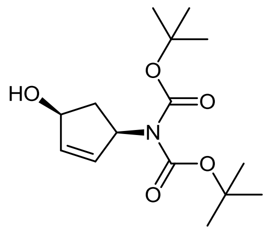

Di-*tert*-butyl [(1*R*,4*S*)-4-hydroxycyclopent-2-en-1-yl]imidodicarbonate (17) [4]

A suspension of sodium di-*tert*-butyl-iminodicarboxylate, previously prepared by reaction of di-*tert*-butyl-iminodicarboxylate (1.195 g, 5.50 mmol) with NaH (60% suspension in mineral oil)(132 mg, 5.50 mmol) in dry THF (18 mL), was cannulated to a room temperature solution of (1*R*,4*S*)-4-hydroxycyclopent-2-enyl acetate (521 mg, 3.67 mmol), PPh_3 (144 mg, 0.55 mmol) and $Pd(PPh_3)_4$ (636 mg, 0.55 mmol) in dry THF/DMF (1:1)(16 mL). The reaction mixture was heated at 50 °C for 1 day, then diluted with MeOH (10 mL) and dry loaded on to silica. Chromatographic purification (Biotage SP4, 50 g cartridge, solvent system: EtOAc/Hex, gradient: 0% 4CV; 0–20% 10CV, 20% 6CV) yielded **17** (463 mg, 42%) as a colourless oil that solidifies upon standing to afford a white solid. ^1H NMR (400 MHz, $CDCl_3$) δ: 1.48 (s, 18H), 1.83 (dt, *J* = 15.1, 2.1 Hz, 1H), 2.62-2.71 (ddd, *J* = 15.1, 9.3, 7.9 Hz, 1H), 3.20 (bs, 1H), 4.58 (app dt, *J* = 7.8, 1.9 Hz, 1H), 5.09 (app dq, *J* = 9.3, 2.3 Hz, 1H), 5.74 (dd, *J* = 5.4, 2.4 Hz, 1H), 6.04 (dt, *J* = 5.4, 2.3 Hz, 1H), ^{13}C NMR (100 MHz, $CDCl_3$) δ: 28.1, 38.9, 60.3, 75.8, 82.9, 131.5, 136.9, 153.4.

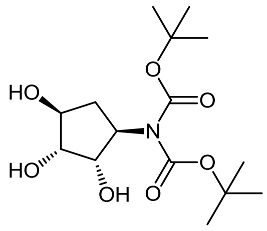

Di-*tert*-butyl [(1*R*,2*S*,3*R*,4*S*)-2,3,4-trihydroxycyclopentyl]imidodicarbonate (18) [5]

A solution of OsO_4 in H_2O (4% w/v, 0.06 mL, 3 mg, 0.01 mmol) was added to a solution of **17** (120 mg, 0.40 mmol) and NMO (141 mg, 1.20 mmol) in a 4:1 mixture acetone/H_2O (10 mL). The solution was stirred at room temperature for 1 day. Saturated aqueous Na_2SO_3 (5 mL) was added and the reaction mixture was stirred for 30 min. The acetone was removed in vacuo and the aqueous layer was extracted with EtOAc (4 × 20 mL). The organic layer was adsorbed on silica gel and concentrated in vacuo. Chromatographic purification (Biotage SP4, 10 g cartridge, solvent system: EtOAc/DCM, gradient: 20% 6CV; 20–80% 8CV; 80% 8CV) yielded **18** (112 mg,

83%) as a colourless oil that solidifies upon standing to afford a white solid. ^{1}H NMR (400 MHz, $CDCl_3$) δ: 1.49 (s, 18H), 1.79-1.85 (m, 1H), 2.58 (ddd, *J* = 14.4, 11.2, 6.3 Hz, 1H), 2.91 (bs, 3H), 3.98 (dd, *J* = 7.1, 4.0Hz, 1H), 4.02 (app dt, *J* = 6.3, 3.1 Hz, 1H), 4.41 (app dt, *J* = 11.1, 6.4 Hz, 1H), 4.56 (dd, *J* = 6.5, 4.8 Hz, 1H); ^{13}C NMR (100 MHz, $CDCl_3$) δ: 28.0, 34.1, 61.9, 74.9, 75.1, 77.3, 83.6, 153.7; HRMS (ESI) calcd for $C_{15}H_{27}O_7NNa$ $[M+Na]^+$: 356.1680, found: 356.1682

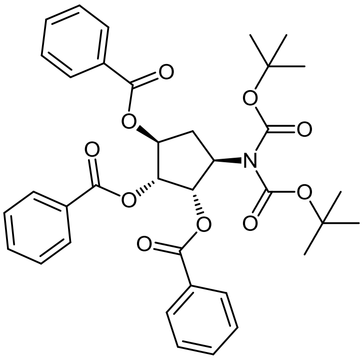

(1*S*,2*R*,3*S*,4*R*)-4-(Bis(*tert*-butoxycarbonyl)amino)cyclopentane-1,2,3-triyl tribenzoate (19)

Benzoyl chloride (0.33 ml, 397 mg, 2.83 mmol) was added dropwise to a stirred solution of **18** (235 mg, 0.71 mmol) in dry pyridine (6 mL) at 0 °C. Left to warn to room temperature and stirred for 1 day. The pyridine was evaporated in vacuo and the residue was partitioned between Et_2O (40 mL) and saturated aqueous $NaHCO_3$ (10 mL). The organic layer was separated and washed with saturated aqueous $NaHCO_3$ (3 × 20 mL) and subsequently adsorbed on silica gel and concentrated in vacuo. Chromatographic purification (Biotage SP4, 50g cartridge, solvent system: Et_2O/Hex, gradient: 0% 6CV; 0–20% 10CV; 20% 6CV) yielded **19** (338 mg, 74%) as a colourless oil that solidifies upon standing to afford a white solid. ^{1}H NMR (400 MHz, $CDCl_3$) δ: 1.51 (s, 18H), 2.25 (ddd, *J* = 12.6, 10.4, 8.8 Hz, 1H), 2.75-2.82 (m, 1H), 5.03 (ddd, *J* = 14.6, 10.0, 5.1 Hz, 1H), 5.61 (app dt, *J* = 7.5, 7.3 Hz, 1H), 5.98-6.05 (m, 2H), 7.29-7.34 (m, 4H), 7.41-7.51 (m, 3H), 7.54-7.63 (m, 2H), 7.90-7.93 (m, 4H), 8.08-8.13 (m, 2H); ^{13}C NMR (100 MHz, $CDCl_3$) δ: 28.1, 30.3, 58.3, 73.7, 74.3, 75.6, 83.3, 128.3, 128.4, 128.5, 129.4, 129.4, 129.6, 129.7, 129.8, 129.9, 133.1, 133.1, 133.2, 152.5, 165.3, 165.4, 166.0; HRMS (ESI) calcd for $C_{36}H_{39}O_{10}NNa$ $[M+Na]^+$: 668.2463, found: 668.2463.

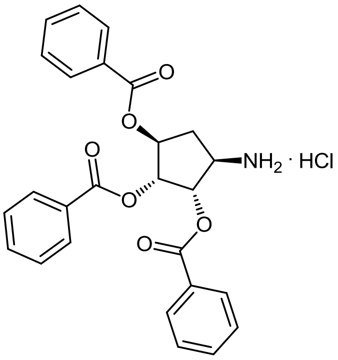

(1*S*,2*R*,3*S*,4*R*)-4-Aminocyclopentane-1,2,3-triyl-tribenzoate hydrochloride (20)

19 (297 mg, 0.46 mmol) was placed in an oven-dry round bottom flask, purged with N_2, cooled to 0 °C and subsequently treated with 4 M HCl in 1,4-dioxane (10 mL). The mixture was let to warm to room temperature, stirred for 16 h and concentrated in vacuo. The residue was triturated with Et_2O (5 × 1 mL) to yield **20** (168 mg, 76%) as a white solid. ^{1}H NMR (400 MHz, $CDCl_3$) δ: 2.01-2.09 (m, 1H), 3.01 (app dt, *J* = 13.9, 8.3 Hz, 1H), 4.07-4.14 (m, 1H), 5.51-5.56 (m, 1H), 5.74 (dd, *J* = 6.4, 6.3 Hz, 1H), 5.82 (dd, *J* = 5.7, 4.7 Hz, 1H), 7.43-7.49 (m, 4H), 7.55-7.59 (m, 2H), 7.63-7.73 (m, 3H), 7.87-7.92 (m, 4H), 8.03-8.05 (m, 2H), 8.66 (bs, 3H); ^{13}C NMR (100 MHz, $CDCl_3$) δ: 31.6, 51.6, 73.6, 74.6, 74.8, 129.0, 129.1, 129.2, 129.2, 129.3, 129.4, 129.8, 129.9, 129.9, 134.2, 164.9, 165.2, 165.4; HRMS (ESI) calcd for $C_{26}H_{24}O_6N$ $[M+H]^+$: 446.1598, found: 446.1599.

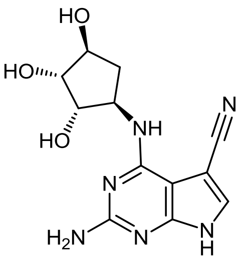

2-Amino-4-((1*R*,2*S*,3*R*,4*S*)-2,3,4-trihydroxycyclopentylamino)-7*H*-pyrrolo[2,3-*d*]pyrimidine-5-carbonitrile (16)

9 (70 mg, 0.25 mmol), **20** (146 mg, 0.30 mmol) and Et_3N (0.08 mL, 61 mg, 0.60 mmol) were suspended in *n*-BuOH (4 mL) and refluxed for 16 h. After cooling to room temperature EtOH (4 mL) and KOH (3 pellets) were added and the reaction mixture was heated at 80 °C for 20 h. Then pH was neutralized using 6 N HCl and subsequently concentrated in vacuo. The remaining solid was suspended in MeOH, adsorbed on silica gel and concentrated in vacuo. Chromatographic purification (Biotage SP4, 10 g cartridge, solvent system: 5% NH_4OH in MeOH/$CHCl_3$, gradient: 2% 6CV; 2–15% 12CV; 10% 15CV) yielded a beige solid which was further

triturated with MeOH (0.2 mL) and Et_2O (2 mL) to yield **16** (24 mg, 33%) as a white solid. $[\alpha]_D^{20}$: -25.7° (c = 0.12, DMSO); Mp: 240-242 °C (d); ^{1}H NMR (400 MHz, DMSO-d_6) δ: 1.27 (ddd, *J* = 13.4, 10.8, 6.7 Hz, 1H), 2.51 (ddd, *J* = 13.8, 8.7, 6.7 Hz, 1H), 3.68 (dd, *J* = 4.7, 2.3 Hz, 1H), 3.81 (m, 1H), 3.93 (dd, *J* = 6.8, 5.0 Hz, 1H), 4.33 (m, 1H), 4.58 (d, *J* = 3.6 Hz, 1H), 4.91 (bs,1H), 4.92 (bs, 1H), 5.70 (d, *J* = 7.5 Hz, 1H), 5.99 (bs, 2H), 7.69 (s, 1H), 11.83 (bs, 1H); ^{13}C NMR (100 MHz, DMSO-d_6) δ: 37.8, 55.2, 74.6, 76.8, 77.6, 94.1, 117.0, 128.5, 129.5, 153.1, 156.2, 160.9; HRMS (ESI) calcd for $C_{12}H_{15}O_3N_6$ $[M+H]^+$: 291.1200, found: 291.1189; HPLC t_R = 6.40 min (Method B)

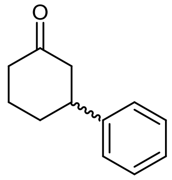

3-Phenylcyclohexanone (25) [6]

To a Schlenk tube were added phenylboronic acid (366 mg, 3.0 mmol), 2-cyclohexenone (288 mg, 3.0 mmol), $Pd(OAc)_2$ (34 mg, 0.15 mmol), 2,2'-bipyridine (34 mg, 0.6 mmol), AcOH (3 mL), THF (1.5 mL), and H_2O (0.3 mL) under N_2. The mixture was stirred and heated at 40 °C for 3 days. After cooling to room temperature, the suspension was diluted with MeOH, adsorbed on silica gel, concentrated in vacuo and eluted through a thick silica pad with Hex (50 mL) then EtOAc/Hex (2:8) (2 × 100 mL) to yield **25** (510 mg, 98%) as a colourless oil. ^{1}H NMR (400 MHz, $CDCl_3$) δ: 1.73–1.89 (m, 2H), 2.06–2.17 (m, 2H), 2.34–2.61 (m, 4H), 2.93-3.03 (m, 1H), 7.21–7.25 (m, 3H), 7.28-7.33 (m, 2H); ^{13}C NMR (100 MHz, $CDCl_3$) δ: 25.5, 32.7, 41.1, 44.7, 48.9, 126.5, 126.6, 128.6, 144.3, 211.0.

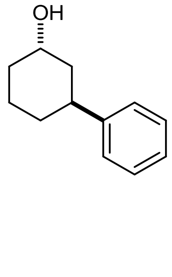

(1*SR*,3*SR*)-3-Phenylcyclohexanol (26) [7]

Under N_2 atmosphere a 2 M solution of **25** in dry THF (322 mg, 1.85 mmol) was added dropwise to a 1 M solution of K-Selectride in THF (3.7 mL, 822 mg, 3.70 mmol) previously cooled to −94 °C (hexane–nitrogen bath). The resulting solution was stirred keeping the temperature between −80 and −90 °C for 2 h and then allowed to warm up to −60 °C, followed by dropwise addition of 50% aqueous KOH (1 mL) and 30% aq. H_2O_2 (1 mL). THF was concentrated in vacuo and the aqueous layer was extracted with EtOAc (3 × 10 mL). The organic layer was dried over anhydrous $MgSO_4$. Chromatographic purification (Manual column, solvent system: EtOAc/Hex, gradient: 0% 60 mL; 10% 200 mL; 20% 300 mL) yielded **26** (261 mg, 80%) as a colourless oil which crystallizes upon standing to an off-white solid. ^{1}H NMR (400 MHz, $CDCl_3$) δ: 1.42-1.72 (m, 5H), 1.79-1.98 (m, 4H), 2.97-3.05 (m, 1H), 4.23-4.24 (m, 1H), 7.17-7.23 (m, 3H), 7.28-7.32 (m, 2H); ^{13}C NMR (100 MHz, $CDCl_3$) δ: 20.5, 32.5, 33.8, 37.6, 40.6, 66.9, 126.0, 127.0, 128.4, 147.1; GC-MS (TIC) calcd for $C_{12}H_{16}O$ [M]: 176.12, found: 176.05.

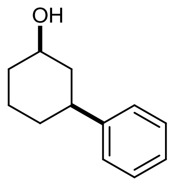

(1*RS*,3*SR*)-3-Phenylcyclohexanol (27) [7]

Under N_2 atmosphere, a solution of **25** (350 mg, 2.01 mmol) in dry Et_2O (6 mL) was added dropwise at 0 °C, to a suspension of $LiAlH_4$ (381 mg, 10.05 mmol) in dry Et_2O (12 mL). The suspension was stirred at 0 °C for 1.5 h, followed by sequential dropwise addition of H_2O (0.5 mL), 15% aq. NaOH (1 mL) and H_2O (0.5 mL). The resulting suspension was stirred at room temperature for 30 minutes. Insoluble matter was filtered off and triturated with Et_2O (4 × 10 mL). The combined organic layers were dried over anhydrous $MgSO_4$. Chromatographic purification (Manual column, solvent system: EtOAc/Hex, gradient: 0% 60 mL; 10% 200 mL; 20% 300 mL) yielded **27** (270 mg, 76%) as a colourless oil which crystallizes upon standing to an off-white solid. ^{1}H NMR (400 MHz, $CDCl_3$) δ: 1.21-1.46 (m, 5H), 1.63 (bs, 1H), 1.81-1.85 (m, 1H), 1.86-1.91 (m, 1H), 2.05-2.10 (m, 1H), 2.16-2.20 (m, 1H), 2.55-2.61 (m, 1H), 3.70-3.77 (m, 1H), 7.20-7.23 (m, 3H), 7.30-7.32 (m,

2H); ^{13}C NMR (100 MHz, $CDCl_3$) δ: 24.5, 33.5, 35.4, 42.8, 43.2, 71.0, 126.1, 126.8, 128.4, 146.2; GC-MS (TIC) calcd for $C_{12}H_{16}O$ [M] : 176.12, found: 176.01.

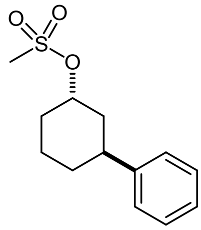

(1*SR*,3*SR*)-3-Phenylcyclohexyl methanesulfonate (28)

Under N_2 atmosphere a solution of methanesulfonyl chloride (0.15 mL, 222 mg, 2.01 mmol) in dry THF (2 mL) was added dropwise at 0 °C, to a solution of **26** (155 mg, 0.88 mmol) and Et_3N (0.32 mL, 232 mg, 2.29 mmol) in dry THF (6 mL). The solution was stirred at room temperature 16 h, followed by addition of MeOH (5 mL). Silica was added to the reaction mixture and then concentrated in vacuo. Chromatographic purification (Biotage SP4, 50 g cartridge, solvent system: EtOAc/Hex, gradient: 0% 4CV; 0–20% 10CV; 20% 8CV) yielded **28** (151 mg, 67%) as a colourless oil. ^1H NMR (400 MHz, $CDCl_3$) δ: 1.49 (app qd, J = 12.5, 3.8 Hz, 1H), 1.58-1.67 (m, 1H), 1.73-1.87 (m, 2H), 1.92-1.97 (m, 1H), 2.10-2.16 (m, 1H), 2.22-2.27 (m, 1H), 2.97-3.03 (m, 2H), 3.04 (s, 3H), 5.15 (app dt, J = 5.1, 2.5 Hz, 1H), 7.18-7.22 (m, 3H), 7.29-7.33 (m, 2H); ^{13}C NMR (100 MHz, $CDCl_3$) δ: 20.6, 30.7, 33.1, 37.7, 38.4, 38.7, 79.5, 126.4, 126.8, 128.6, 145.7.

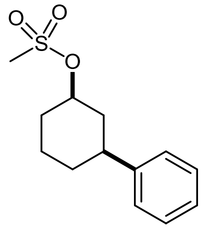

(1*RS*,3*SR*)-3-Phenylcyclohexyl methanesulfonate (29)

Under N_2 atmosphere, a solution of methanesulfonyl chloride (0.65 mL, 966 mg, 8.43 mmol) in dry THF (8 mL) was added dropwise at 0 °C, to a solution of **27** (675 mg, 3.83 mmol) and Et_3N (1.4 mL, 1.0 g, 9.96 mmol) in dry THF (4 mL). The solution was stirred at room temperature 16 h, followed by addition of MeOH (5 mL). Silica was added to the reaction mixture and then concentrated in vacuo. Chromatographic purification (Biotage SP4,

50 g cartridge, solvent system: EtOAc/Hex, gradient: 0% 4CV; 0–20% 10CV; 20% 8CV) yielded **29** (620 mg, 64%) as a colourless oil which crystallizes upon standing to an off-white solid. ^{1}H NMR (400 MHz, $CDCl_3$) δ: 1.37 (app qd, *J* = 12.4, 3.3 Hz, 1H), 1.44-1.62 (m, 2H), 1.72 (app q, *J* = 12.0 Hz, 1H), 1.82-1.87 (m, 1H), 1.94-1.99 (m, 1H), 2.22-2.26 (m, 1H), 2.32-2.38 (m, 1H), 2.64 (app tt, *J* = 12.3, 3.3 Hz, 1H), 3.00 (s, 3H), 4.72-4.79 (app tt, *J* = 11.1, 4.6 Hz, 1H), 7.18-7.23 (m, 3H), 7.28-7.32 (m, 2H); ^{13}C NMR (100 MHz, $CDCl_3$) δ: 24.3, 32.77, 32.79, 38.9, 40.4, 42.7, 81.6, 126.4, 126.9, 128.7, 145.1.

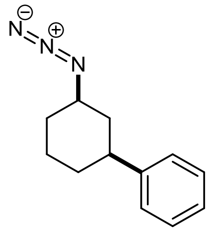

((1*SR*,3*RS*)-3-Azidocyclohexyl)benzene (30)

NaN_3 (395 mg, 6.07 mmol) was added to a solution of **28** (386 mg, 1.52 mmol) in DMF (3 mL). The reaction mixture was stirred at 80 °C for 2 days. The reaction mixture was allowed to cool to room temperature, brine (10 mL) was added and the aqueous phase was washed with Hex (4 × 10 mL). Silica was added to the organic layer and then concentrated in vacuo. Chromatographic purification (Biotage SP4, 50 g cartridge, solvent system: EtOAc/Hex, gradient: 0% 10CV; 0–10% 5CV) yielded **30** (170 mg, 56%) as a colourless oil. ^{1}H NMR (400 MHz, $CDCl_3$) δ: 1.30-1.55 (m, 4H), 1.85-1.89 (m, 1H), 1.94-1.98 (m, 1H), 2.06-2.09 (m, 1H), 2.17-2.20 (m, 1H), 2.59 (app tt, *J* = 12.1, 3.3 Hz, 1H), 3.41 (app tt, *J* = 11.5, 4.0 Hz, 1H), 7.19-7.23 (m, 3H), 7.29-7.33 (m, 2H); ^{13}C NMR (100 MHz, $CDCl_3$) δ: 24.3, 31.0, 32.6, 38.9, 42.6, 59.8, 125.9, 126.3, 128.1, 145.2; LRMS (ESI) calcd for $C_{12}H_{15}N$ $[M-N_2]^+$: 173.1, found: 174.2.

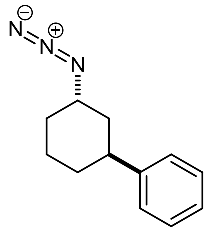

((1*SR*,3*SR*)-3-Azidocyclohexyl)benzene (31)

NaN_3 (351 mg, 5.397 mmol) was added to a solution of **29** (343 mg, 1.35 mmol) in DMF (3 mL). The reaction mixture was stirred at 80 °C for 2 days. The reaction mixture was allowed to cool to room temperature, brine (10 mL) was added and the aqueous phase was washed with Hex (4 × 10 mL). Silica was added to the organic layer and then concentrated in vacuo. Chromatographic purification (Biotage SP4, 50 g cartridge, solvent system: EtOAc/Hex, gradient: 0% 10CV; 0–10% 5CV) yielded **31** (182 mg, 67%) as a colourless oil. ^1H NMR (400 MHz, $CDCl_3$) δ: 1.48 (app qd, *J* = 12.4, 4.4 Hz, 1H), 1.54-1.62 (m, 1H), 1.66-1.77 (m, 3H), 1.90-1.93 (m, 2H), 1.99-2.05 (m, 1H), 2.89 (app tt, *J* = 12.2, 3.3 Hz, 1H), 4.06 (app dt, *J* = 6.2, 3.1 Hz, 1H), 7.19-7.22 (m, 3H), 7.29-7.33 (m, 2H); ^{13}C NMR (100 MHz, $CDCl_3$) δ: 21.1, 29.3, 33.3, 37.4, 38.2, 58.2, 126.2, 126.9, 128.5, 146.3; LRMS (ESI) calcd for $C_{12}H_{15}N$ $[M-N_2]^+$: 173.1, found: 174.0.

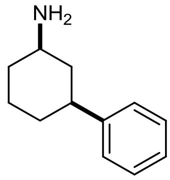

(1*RS*,3*SR*)-3-Phenylcyclohexanamine (23)

A suspension of **30** (177 mg, 0.88 mmol), 10% Pd/C (177 mg) and HCO_2NH_4 (277 mg, 4.40 mmol) in MeOH (10 mL) was refluxed for 1.5 h. After cooling down to room temperature, the reaction mixture was diluted in EtOAc (20 mL), filtered through a thick pad of Celite®, washed with EtOAc (2 × 5 mL) and concentrated in vacuo to yield **23** (145 mg, 94%) as a yellow oil. FTIR (neat): 3420.4, 3360.3, 2920.1, 2850.3, 1594.6, 1498.5, 1430.6, 703.4, 723.8; ^1H NMR (400 MHz, $CDCl_3$) δ: 1.09 (app qd, *J* = 12.2, 3.4 Hz, 1H), 1.22-1.47 (m, 3H), 1.51 (bs, 2H), 1.83-1.94 (m, 3H), 2.00-2.04 (m, 1H), 2.57 (app tt, *J* = 12.1, 3.3 Hz, 1H), 2.80 (app tt, *J* = 11.1, 3.8 Hz, 1H), 7.18-7.21 (m, 3H), 7.27-7.31 (m, 2H); ^{13}C NMR (100 MHz, $CDCl_3$) δ: 24.9, 33.1, 35.9, 42.9, 44.1, 50.5, 125.5, 126.3, 127.9, 146.4; HRMS (ESI) calcd for $C_{12}H_{18}N$ $[M+H]^+$: 176.1434, found: 176.1433.

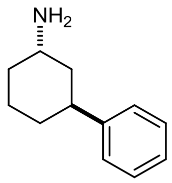

(1*SR*,3*SR*)-3-Phenylcyclohexanamine (24)

A suspension of **31** (146 mg, 0.88 mmol), 10% Pd/C (146 mg) and HCO_2NH_4 (229 mg, 3.63 mmol) in MeOH (10 mL) was refluxed for 1.5 h. After cooling down to room temperature, the reaction mixture was diluted in EtOAc (20 mL), filtered through a thick pad of Celite®, washed with EtOAc (2 × 5 mL) and concentrated in vacuo to yield **24** (117 mg, 92%) as a yellow oil. FTIR (neat): 3418.2, 3341.2, 2921.1, 2855.2, 1595.6, 1494.2, 1431.1, 706.4, 728.9; ^{1}H NMR (400 MHz, $CDCl_3$) δ: 1.39 (app qd, *J* = 12.2, 3.3 Hz, 1H), 1.44-1.52 (m, 3H), 1.63 (app dd, *J* = 8.2, 3.4 Hz, 2H), 1.70-1.85 (m, 2H), 2.23 (bs, 2H), 2.94-3.02 (m, 1H), 3.22 (app dt, *J* = 6.7, 3.3 Hz, 1H), 7.12-7.17 (m, 1H), 7.18-7.22 (m, 2H), 7.25-7.29 (m, 2H); ^{13}C NMR (100 MHz, $CDCl_3$) δ: 20.4, 32.9, 34.2, 37.0, 41.1, 46.1, 126.1, 127.3, 128.7, 147.9; HRMS (ESI) calcd for $C_{12}H_{18}N$ $[M+H]^+$: 176.1434, found: 176.1435.

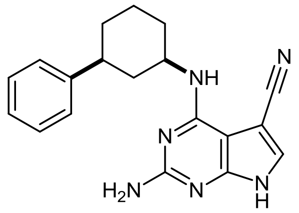

2-Amino-4-(1*RS*,3*SR*-phenylcyclohexylamino)-7*H*-pyrrolo[2,3-*d*]pyrimidine-5-carbonitrile (21)

9 (135 mg, 0.49 mmol), **23** (102 mg, 0.58 mmol) and Et_3N (0.07 mL, 49 mg, 0.49 mmol) were suspended in *n*-BuOH (4 mL) and refluxed for 16 h. After cooling to room temperature EtOH (4 mL) and KOH (2 pellets) were added and the reaction mixture was heated at 80 °C for 20 h. Then pH was neutralized using 6 N HCl and subsequently concentrated in vacuo. The remaining solid was suspended in MeOH, adsorbed on silica gel and concentrated in vacuo. Chromatographic purification (Biotage SP4, 50 g cartridge, solvent system: EtOAc/Hex, gradient: 60% 6CV; 60–100% 10CV; 100% 4CV) yielded a beige solid that was further triturated with MeOH (0.2 mL) to afford **21** (80 mg, 50%) as a white solid. Mp: 254-256 °C (d); ^{1}H NMR (400 MHz, DMSO-d_6) δ: 1.29-1.56 (m, 4H), 1.76-1.79 (m, 1H), 1.86-1.89 (m, 1H), 2.06-2.15 (m, 2H), 2.63-2.69 (m, 1H), 4.13-4.20 (m, 1H), 5.39 (d, *J* = 5.4 Hz, 1H), 5.96 (bs, 2H), 7.16-7.19 (m, 1H), 7.25-7.31 (m, 4H), 7.62 (s, 1H), 10.95 (bs, 1H);

^{13}C NMR (100 MHz, DMSO-d$_6$) δ: 26.0, 33.3, 34.3, 41.2, 44.2, 50.3, 83.6, 95.7, 117.4, 126.9, 127.6, 128.2, 129.2, 147.5, 153.7, 157.0, 162.8; HRMS (ESI) calcd for $C_{19}H_{21}N_6$ $[M+H]^+$: 333.1822, found: 333.1819; HPLC t_R = 13.03 min (Method A)

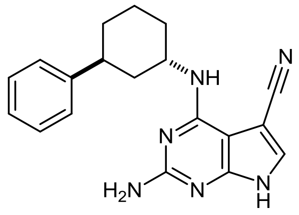

2-Amino-4-(1*SR*,3*RS*-3-phenylcyclohexylamino)-7*H*-pyrrolo[2,3-d]pyrimidine-5-carbonitrile (22)

9 (160 mg, 0.58 mmol), **24** (121 mg, 0.69 mmol) and Et_3N (0.08 mL, 58 mg, 0.58 mmol) were suspended in *n*-BuOH (4 mL) and refluxed for 16 h. After cooling to room temperature EtOH (4 mL) and KOH (2 pellets) were added and the reaction mixture was heated at 80 °C for 20 h. Then pH was neutralized using 6 N HCl and subsequently concentrated in vacuo. The remaining solid was suspended in MeOH, adsorbed on silica gel and concentrated in vacuo. Chromatographic purification (Biotage SP4, 50 g cartridge, solvent system: MeOH/DCM, gradient: 0% 6CV; 0–5% 8CV; 5% 8CV) yielded a beige solid that was further triturated with MeOH (0.5 mL) to afford **22** (72 mg, 38%) as a white solid. Mp: 270-272 °C (d); ^{1}H NMR (400 MHz, DMSO-d$_6$) δ: 1.50-1.74 (m, 4H), 1.78-1.86 (m, 2H), 1.91-1.93 (m, 1H), 2.02-2.06 (m, 1H), 2.77 (app dt, *J* = 11.8, 3.1 Hz, 1H), 4.44-4.52 (m, 1H), 5.69 (d, *J* = 7.1 Hz, 1H), 5.99 (bs, 2H), 7.16-7.20 (m, 1H), 7.22-7.24 (m, 2H), 7.28-7.31 (m, 2H), 7.71 (s, 1H), 11.85 (bs, 1H); ^{13}C NMR (100 MHz, DMSO-d$_6$) δ: 21.4, 29.8, 33.6, 37.8, 38.7, 45.5, 81.9, 95.3, 118.1, 126.6, 127.3, 128.5, 128.9, 146.8, 153.4, 156.1, 161.7; HRMS (ESI) calcd for $C_{19}H_{21}N_6$ $[M+H]^+$: 333.1822, found: 333.1818; HPLC t_R = 13.05 min (Method A).

References

1. Gangjee, A.; Vidwans, A.; Elzein, E.; McGuire, J. J.; Queener, S. F.; Kisliuk, R. L., *J. Med. Chem.* **2001,** *44* (12), 1993-2003.
2. Brueckl, T.; Klepper, F.; Gutsmiedl, K.; Carell, T., *Org. Biomol. Chem.* **2007,** *5*, 3821-3825.
3. Bond, C. W.; Cresswell, A. J.; Davies, S. G.; Fletcher, A. M.; Kurosawa, W.; Lee, J. A.; Roberts, P. M.; Russell, A. J.; Smith, A. D.; Thomson, J. E., *J. Org. Chem.* **2009,** *74* (17), 6735-6748.
4. Dauvergne, J.; Happe, A. M.; Jadhav, V.; Justice, D.; Matos, M.-C.; McCormack, P. J.; Pitts, M. R.; Roberts, S. M.; Singh, S. K.; Snape, T. J.; Whittall, J., *Tetrahedron* **2004,** *60* (11), 2559-2567.

5. Springthorpe, B.; Bailey, A.; Barton, P.; Birkinshaw, T. N.; Bonnert, R. V.; Brown, R. C.; Chapman, D.; Dixon, J.; Guile, S. D.; Humphries, R. G.; Hunt, S. F.; Ince, F.; Ingall, A. H.; Kirk, I. P.; Leeson, P. D.; Leff, P.; Lewis, R. J.; Martin, B. P.; McGinnity, D. F.; Mortimore, M. P.; Paine, S. W.; Pairaudeau, G.; Patel, A.; Rigby, A. J.; Riley, R. J.; Teobald, B. J.; Tomlinson, W.; Webborn, P. J. H.; Willis, P. A., *Bioorg. Med. Chem. Lett.* **2007,** *17* (21), 6013-6018.
6. Lu, X.; Lin, S., *J. Org. Chem.* **2005,** *70* (23), 9651-9653.
7. Kawamura, S.; Kawabata, T.; Ishizuka, K.; Nakamura, M., *Chem. Comm.* **2012,** *48* (75), 9376-9378.

Copies of ^1H and ^{13}C NMR spectra and HPLC UV traces

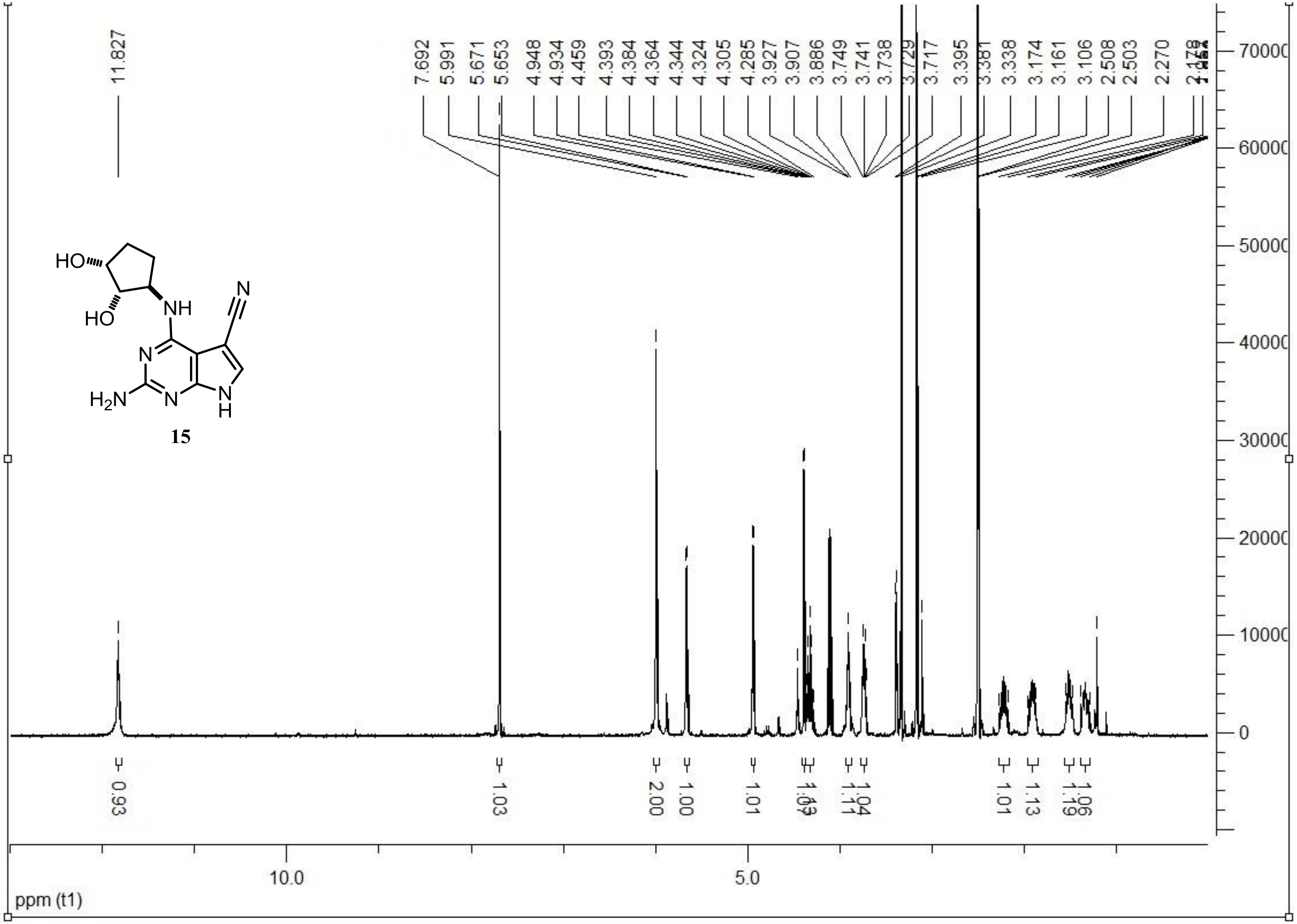

15

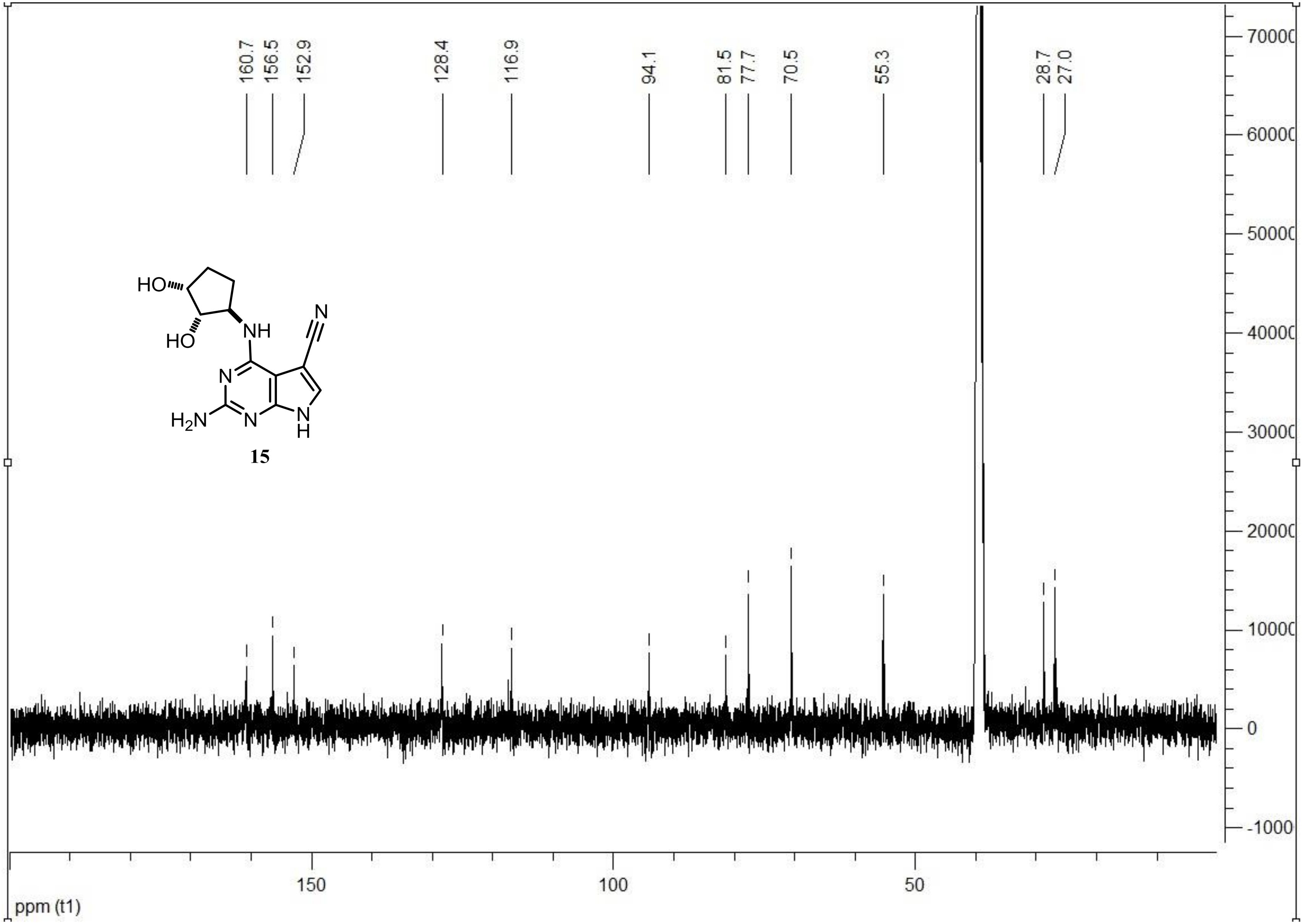
160.7
156.5
152.9
128.4
116.9
94.1
81.5
77.7
70.5
55.3
28.7
27.0
HO
HO
NH
N
N
N
H₂N
N
H
15
70000
60000
50000
40000
30000
20000
10000
0
-1000
150
100
50
ppm (t1)

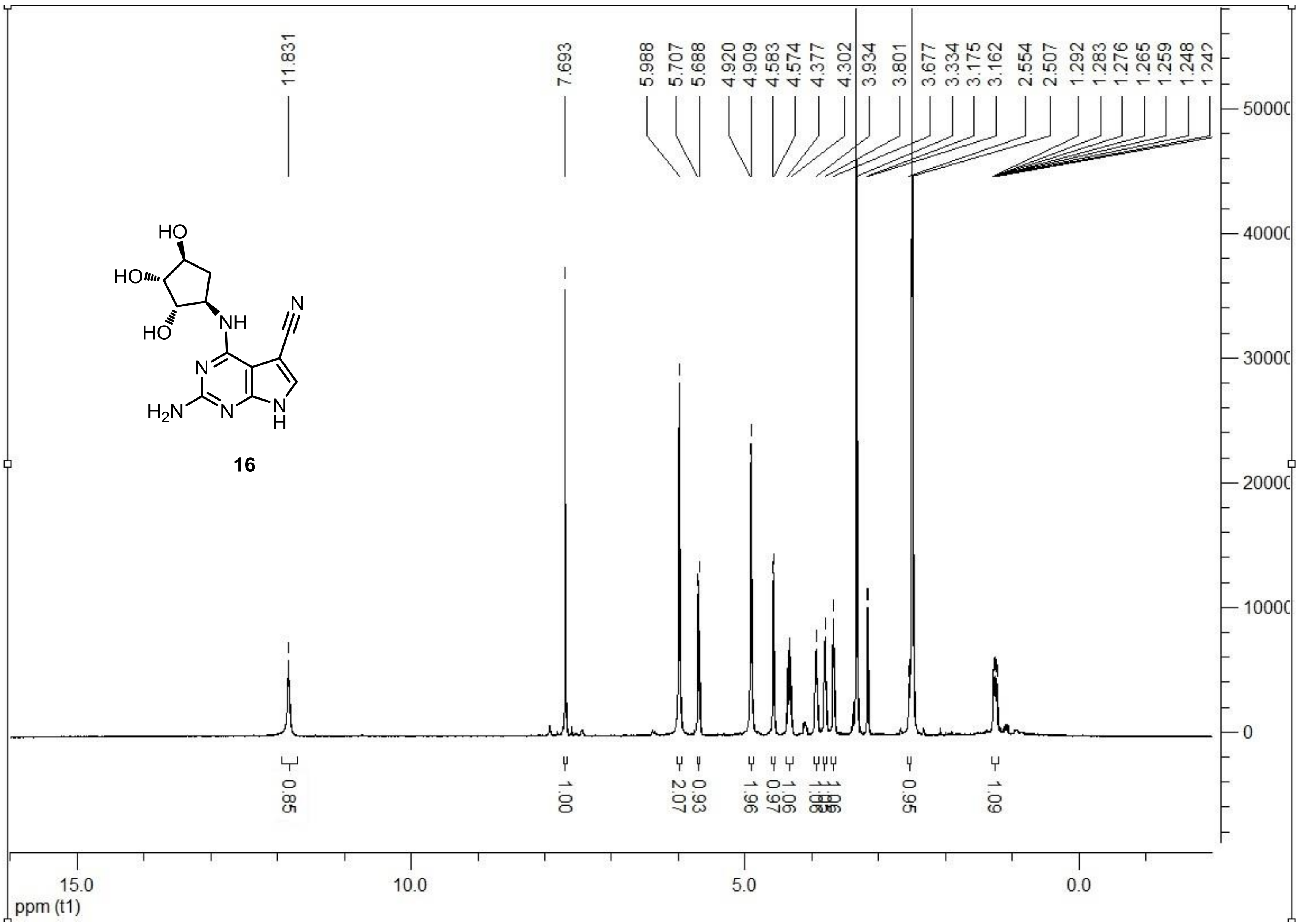
11.831
7.693
5.988
5.707
5.688
4.920
4.909
4.583
4.574
4.377
4.302
3.934
3.801
3.677
3.334
3.175
3.162
2.554
2.507
1.292
1.283
1.276
1.265
1.259
1.248
1.242
50000
40000
30000
20000
10000
0
0.85
1.00
2.07
0.93
1.96
0.97
1.06
0.95
1.09
15.0
10.0
5.0
0.0
ppm (t1)
HO
HO
HO
NH
N
N
N
H2N
N
H

16

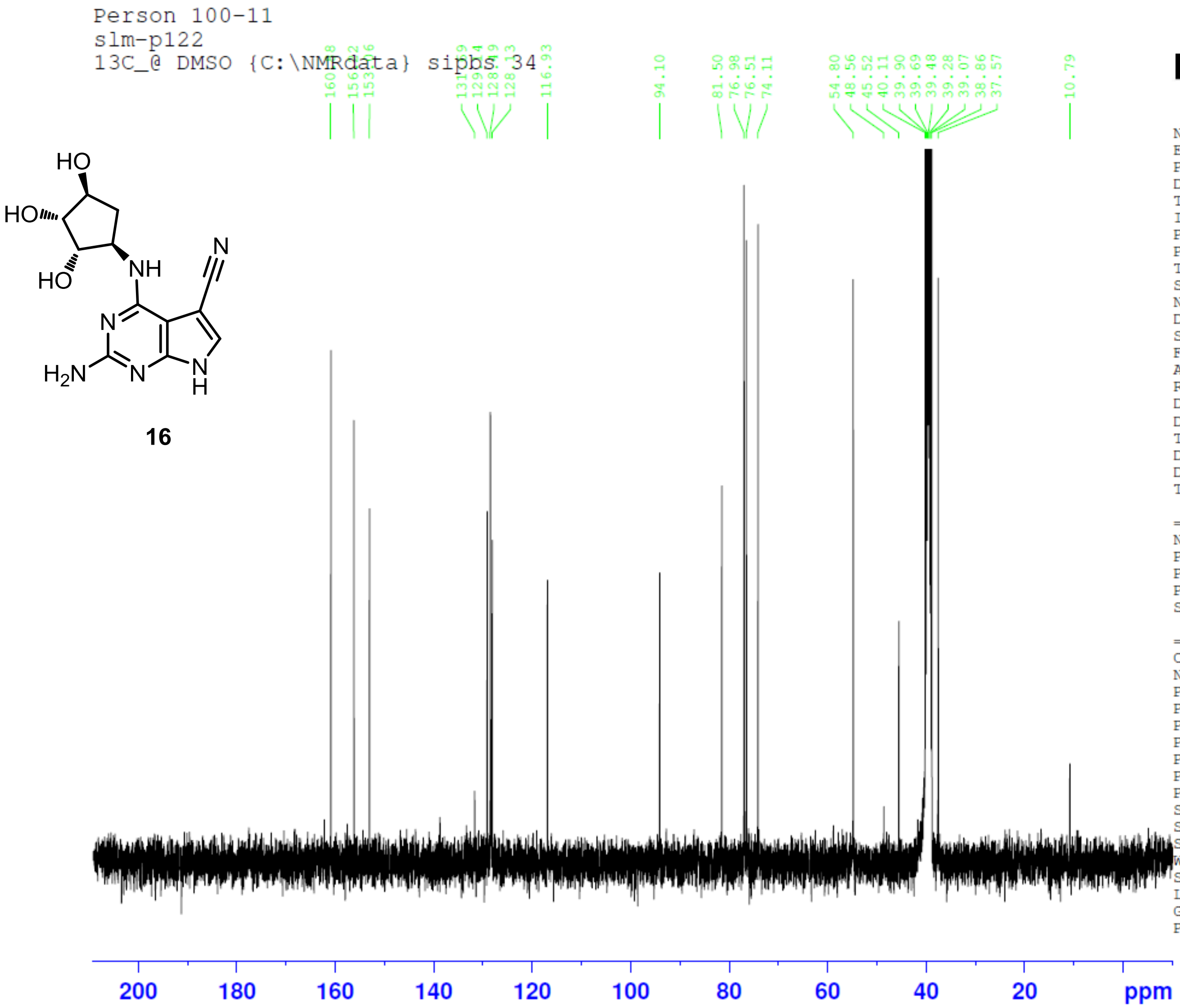

NAME	D144884	
EXPNO	1	
PROCNO	1	
Date_	20110506	
Time	22.00	
INSTRUM	spect	
PROBHD	5 mm QNP 1H/13	
PULPROG	zgpg30	
TD	16384	
SOLVENT	DMSO	
NS	5000	
DS	4	
SWH	22058.824	Hz
FIDRES	1.346364	Hz
AQ	0.3714207	sec
RG	2050	
DW	22.667	usec
DE	6.50	usec
TE	296.2	K
D1	0.69999999	sec
D11	0.03000000	sec
TD0	1	

======== CHANNEL f1 ========

NUC1	13C	
P1	7.40	usec
PL1	-3.00	dB
PL1W	67.25932312	W
SFO1	100.6228298	MHz

======== CHANNEL f2 ========

CPDPRG2	waltz16	
NUC2	1H	
PCPD2	80.00	usec
PL2	-4.00	dB
PL12	15.17	dB
PL13	17.00	dB
PL2W	19.93825150	W
PL12W	0.24137211	W
PL13W	0.15837516	W
SFO2	400.1316005	MHz
SI	32768	
SF	100.6128193	MHz
WDW	EM	
SSB	0	
LB	1.00	Hz
GB	0	
PC	1.40	

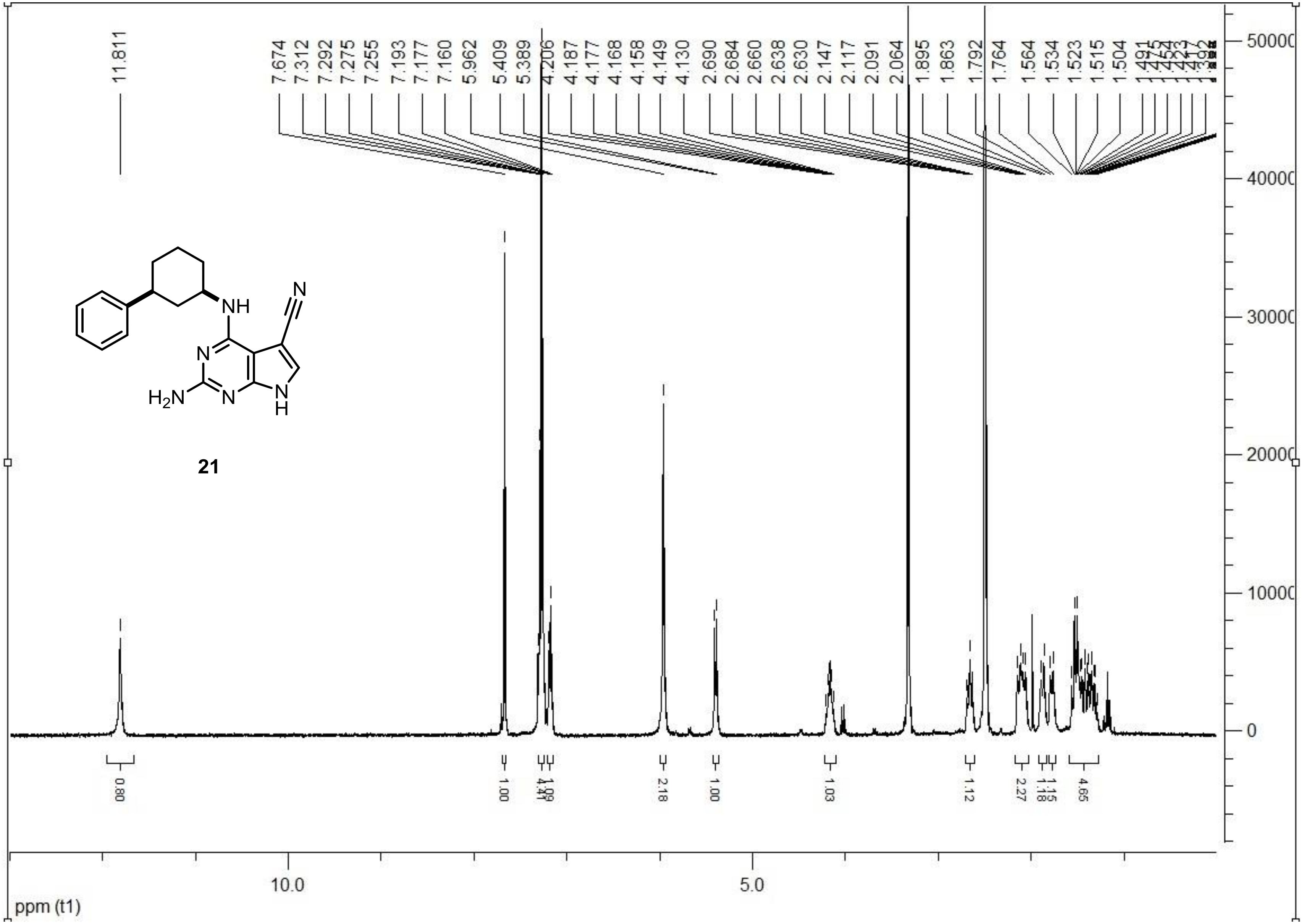

11.811
7.674
7.312
7.292
7.275
7.255
7.193
7.177
7.160
5.962
5.409
5.389
4.206
4.187
4.177
4.168
4.158
4.149
4.130
2.690
2.684
2.660
2.638
2.630
2.147
2.117
2.091
2.064
1.895
1.863
1.792
1.764
1.564
1.534
1.523
1.515
1.504
1.491
0.80
1.00
2.18
1.00
1.03
1.12
2.27
1.18
1.15
4.65
50000
40000
30000
20000
10000
0
10.0
5.0
ppm (t1)

21

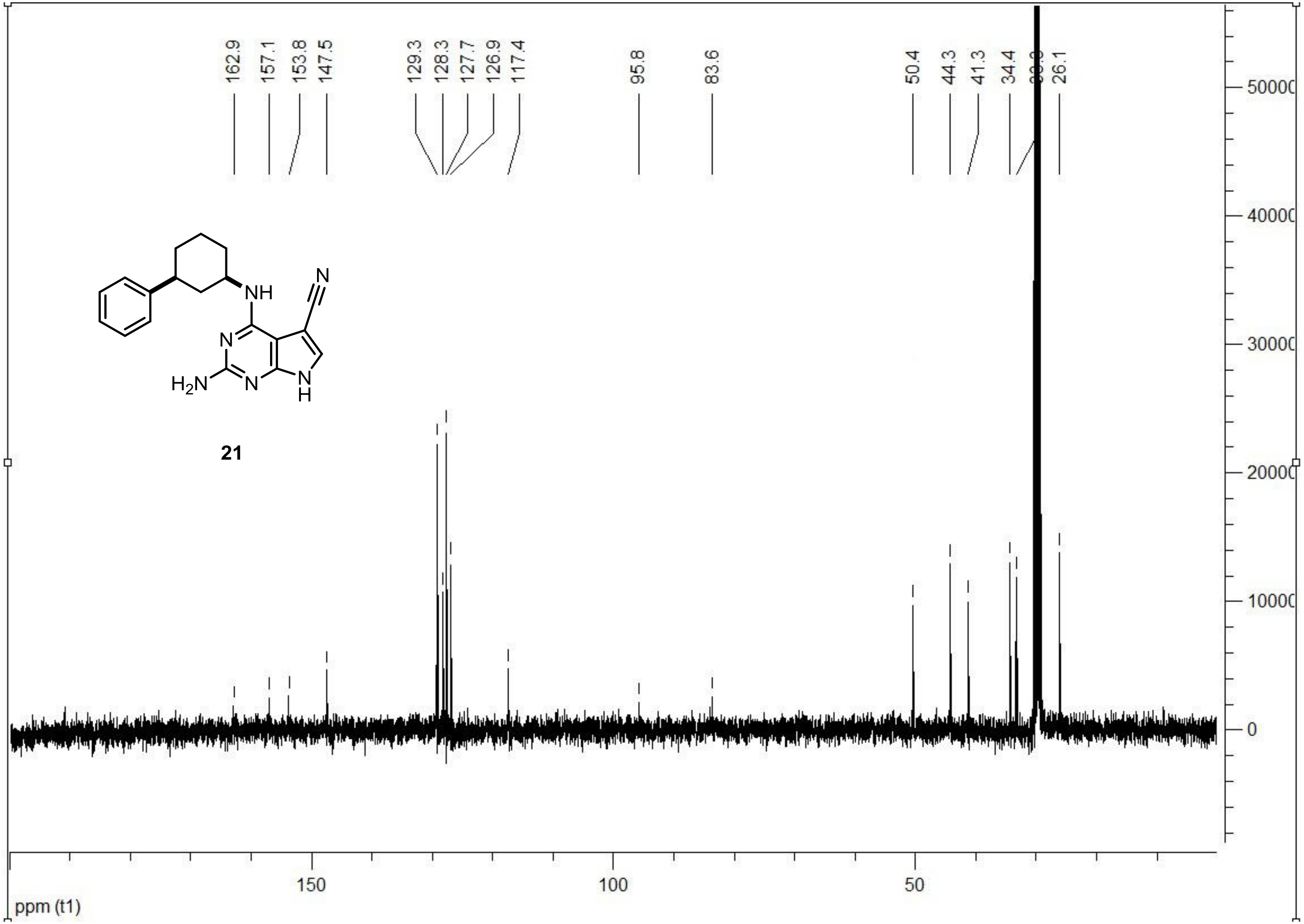
162.9
157.1
153.8
147.5
129.3
128.3
127.7
126.9
117.4
95.8
83.6
50.4
44.3
41.3
34.4
26.1
NH
N
N
H_2N
N
H
21
150
100
50
ppm (t1)
50000
40000
30000
20000
10000
0

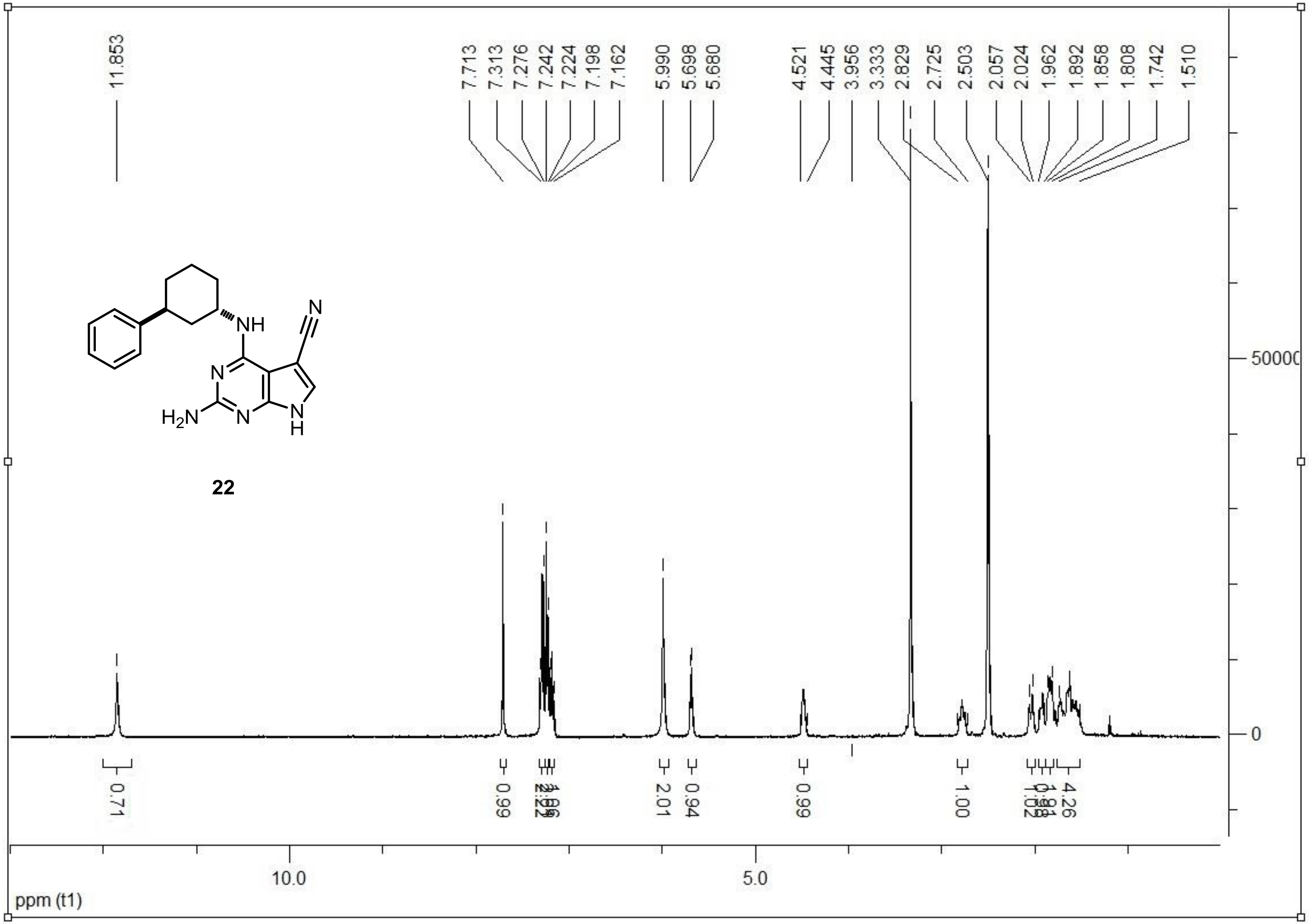
11.853
7.713
7.313
7.276
7.242
7.224
7.198
7.162
5.990
5.698
5.680
4.521
4.445
3.956
3.333
2.829
2.725
2.503
2.057
2.024
1.962
1.892
1.858
1.808
1.742
1.510
NH
N
H_2N
N
N
H
N
22
50000
0
0.71
0.99
2.01
0.94
0.99
1.00
4.26
10.0
5.0
ppm (t1)

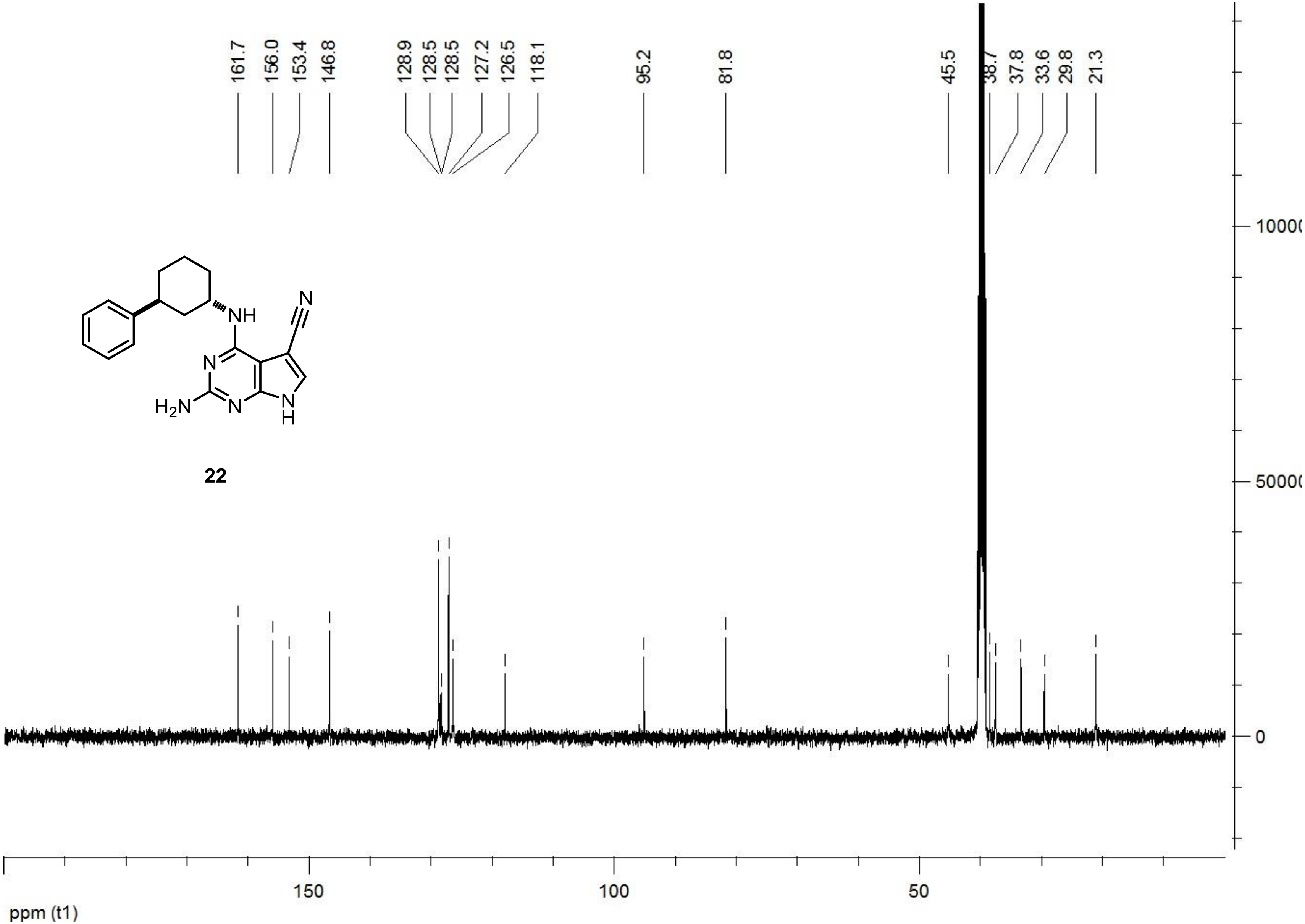
161.7
156.0
153.4
146.8
128.9
128.5
128.5
127.2
126.5
118.1
95.2
81.8
45.5
38.7
37.8
33.6
29.8
21.3
NH
N
H2N
N
N
H
N
100000
50000
0
150
100
50
ppm (t1)

22

Peak #	RetTime [min]	Type	Width [min]	Area [mAU*s]	Height [mAU]	Area %
1	2.297	MM	0.0475	6.01752	2.11248	0.8052
2	4.471	MM	0.1084	741.31653	113.95839	99.1948

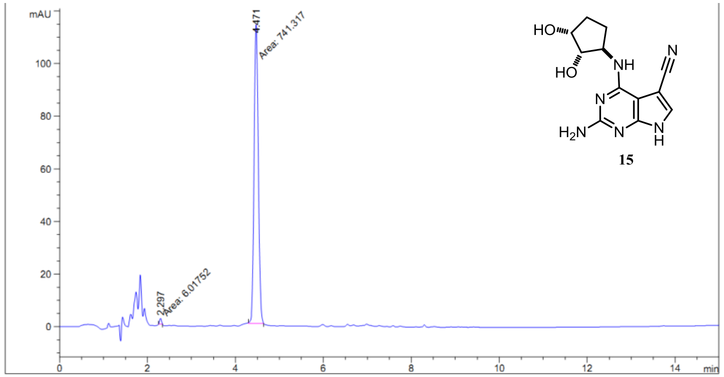

15

Peak #	RetTime [min]	Type	Width [min]	Area [mAU*s]	Height [mAU]	Area %
1	2.105	MM	0.0922	78.33704	14.15373	1.5391
2	4.080	MM	0.1137	44.86591	6.57890	0.8815
3	6.399	VV	0.0910	4889.03906	803.13287	96.0532
4	7.754	MM	0.0538	40.36665	12.50004	0.7931
5	8.375	MM	0.0499	37.31915	12.45732	0.7332

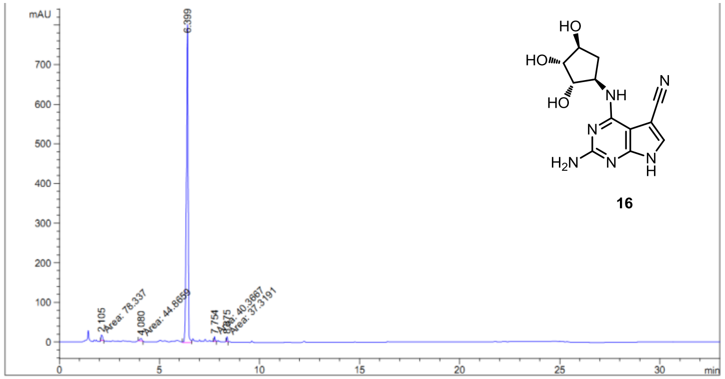

16

Peak #	RetTime [min]	Type	Width [min]	Area [mAU*s]	Height [mAU]	Area %
1	9.576	MM	0.0577	6.86780	1.98521	0.7253
2	11.317	MM	0.0527	2.89513	9.14746e-1	0.3057
3	11.756	MM	0.0590	15.08131	4.25965	1.5927
4	13.031	MM	0.0761	922.06757	201.84230	97.3763

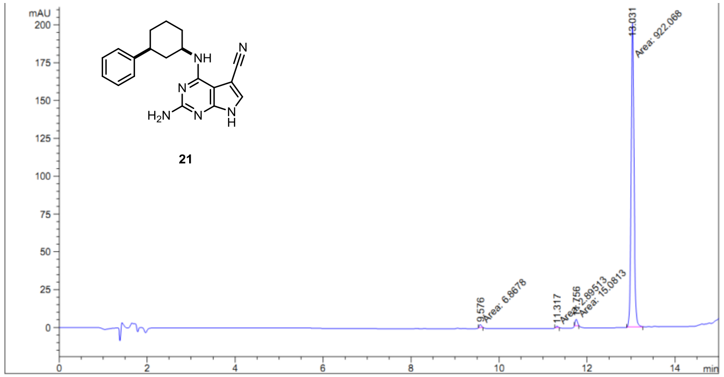

21

Peak #	RetTime [min]	Type	Width [min]	Area [mAU*s]	Height [mAU]	Area %
1	10.425	MM	0.0524	4.67118	1.48640	1.0110
2	13.052	MM	0.1410	457.38342	54.05390	98.9890

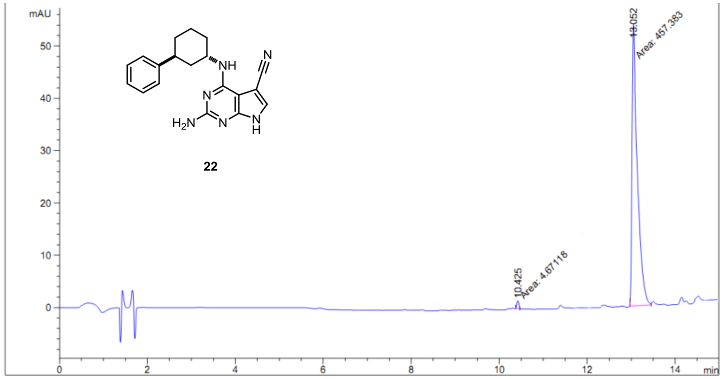

22
